# Human Fibroblast Reprogramming to Pluripotent Stem Cells Regulated by the miR19a/b-PTEN Axis

**DOI:** 10.1371/journal.pone.0095213

**Published:** 2014-04-16

**Authors:** Xiaoping He, Yang Cao, Lihua Wang, Yingli Han, Xiuying Zhong, Guixiang Zhou, Yongping Cai, Huafeng Zhang, Ping Gao

**Affiliations:** 1 Innovation Center for Cell Biology, School of Life Science, University of Science and Technology of China, Hefei, China; 2 Center for Reproductive Medicine, Anhui Provincial Hospital Affiliated to Anhui Medical University, Hefei, China; 3 Department of Pathology, School of Medicine, Anhui University, Hefei, China; Johns Hopkins Univ. School of Medicine, United States of America

## Abstract

Induction of pluripotent stem cells (iPSC) by defined transcription factors is the recognized canonical means for somatic reprogramming, however, it remains incompletely understood how individual transcription factors affect cell fate decisions during the reprogramming process. Here, we report induction of fibroblast reprogramming by various transcriptional factors is mediated by a miR19a/b-PTEN axis. cMyc, one of the four Yamanaka factors known to stimulate both somatic cell reprogramming and tumorigenesis, induced the expression of multiple mircoRNAs, miR-17∼92 cluster in particular, in the early stage of reprogramming of human fibroblasts. Importantly, miR-17∼92 cluster could greatly enhance human fibroblast reprogramming induced by either the four Yamanaka factors (Oct4, Sox2, Klf4, and cMyc, or 4F) or the first three transcriptional factors (Oct4, Sox2, and Klf4, or 3F). Among members of this microRNA cluster, miR-19a/b exhibited the most potent effect on stimulating fibroblst reprogramming to iPSCs. Additional studies revealed that miR-19a/b enhanced iPSC induction efficiency by targeted inhibition of phosphatase and tensin homolog (PTEN), a renowned tumor suppressor whose loss-of-function mutations were found in multiple human malignancies. Our results thus demonstrate an important role of miR-19a/b-PTEN axis in the reprogramming of human fibroblasts, illustrating that the somatic reprogramming process and its underlying regulation pathways are intertwined with oncogenic signaling in human malignancies.

## Introduction

The past few years have seen a significant advance in reprogramming study following Shinya Yamanaka's seminal discovery in 2006 [1]. Initially, different cocktails of transcription factors were described for pluripotency induction [2], [3], [4]. Subsequently, certain epigenetic regulators capable of altering chromatin state through histone modifications or DNA methylation were found to participate in somatic cell reprogramming [5], [6]. More recently, several studies have shown that mouse fibroblasts can be reprogrammed into iPSCs using nuclear factors that control lineage specification [7], [8]. Surprisingly, it seems that none of the initial four Yamanaka factors is essential for reprogramming induction [9]. Nowadays there is no doubt that pluripotency could be induced by various protocols although pluripotency induction using defined transcription factors remains the recognized canonical means. Nevertheless, it remains largely unclear how these transcription factors facilitate the reprogramming process.

Of the four Yamanaka factors initially used for human somatic reprogramming, *cMyc* is the most intriguing one in that its multifaceted functions have been extensively studied in various homeostatic and diseased contexts [10]. *cMyc* is a proto-oncogene whose deregulated expression was observed in 30–50% of all human malignancies [11]. More recently, *cMyc* has been documented to regulate approximately 10–15% of all human genes [12]. Although cMyc is one of the four Yamanaka factors that induce somatic cell reprogramming [1], iPSC can also be generated without cMyc, albeit with much lower efficiency [13], [14]. Moreover, cMyc can induce the expression of many more genes than other Yamanaka factors, suggesting that cMyc is likely to be more widely involved in reprogramming process [15], [16]. Hence, it is essential and strategically appealing to better understand cMyc's role in reprogramming of human somatic cells.

The path to reprogramming induced by Yamanaka factors involves multiple steps and various regulatory mechanisms [16], [17], [18], [19]. It is reported that some mircoRNAs (miRNAs) are involved in this path and hence can regulate the outcome of reprogramming. *Judson et al.* claim that ES cell-specific cell cycle regulating (ESCC) miRNAs miR-291-3p, -294, -295, and -302d enhance reprogramming in mouse system [20]. *Anokye-Danso et al.* and *Miyoshi et al.* have reported that miRNA cluster 302–367 can reprogram iPSCs independent of transcription factors [21], [22]. Recent studies also show that miR-29b, miR-138, and several other miRNAs enhance reprogramming, whereas miR-34 and let-7 act as a barrier of reprogramming [23], [24], [25], [26]. *cMyc* is the first oncogene reported to regulate miRNAs in tumor cells [27]. In a human B lymphoma system, we have recently documented that cMyc regulates miRNAs mir-23a/b, which controls glutaminase expression and glutamine metabolism in tumor cells [28]. Although cMyc-mediated gene expression has previously been addressed during somatic reprogramming process [14], it is not clear what miRNAs are regulated by cMyc in this process and what roles they might play.

Looking back carefully at the tremendous efforts made in the past several years to understand the process of reprogramming and its underlying mechanisms, it is intriguing to notice the similarity between oncogenic and reprogramming processeses. Many oncogenes and their downstream effectors such as *Ras* and *SV40* T antigen are found to facilitate reprogramming [29], [30], whereas tumor suppressors like *P53* and *let-7* repressing it [26], [31]. Gaining insight into this similarity may not only be important for understanding the reprogramming mechanisms but also have critical implications for safety control of iPSCs, taking into account of the fact that many iPSC-generated mice displayed higher tumorigenic tendency [32]. Here, we report that cMyc via its target oncomirs mir-17-92 cluster can significantly enhance human somatic reprogramming. Moreover, our data demonstrate that miR-19a and miR-19b, which are oncogenic in human malignancies [27], are the most potent to stimulate induction of iPSCs. Most interestingly, we identified PTEN, a renowned tumor suppressor, as a target that facilitates miR-19a/b-mediated human cell reprogramming. Taken together, the results of the present study establish for the first time the pivot role of mir19a/b-PTEN axis in regulating human somatic cell reprogramming, revealing interestingly that the process of human reprogramming and its underlying regulation pathways are complicatedly intertwined with oncogenic process in human malignancies.

## Materials and Methods

### Cell culture and reagents

Human fibroblast IMR90 cells were purchased from ATCC and maintained in Dulbecco's modified eagle medium (DMEM, Corning) containing 10% fetal bovine serum (FBS, Invitrogen) and 1×10^−4^ M nonessential amino acids (NEAA, Invitrogen). HEK293T cells were maintained in DMEM containing 10% FBS (Hyclone). Human iPSCs were generated on mitomycin C-treated MEFs (as feeder cells) in DMEM/F12 (Invitrogen) containing 20% Knockout Serum Replacement (KSR, Invitrogen), 2 mM L-glutamine (Invitrogen), 1×10^−4^ M NEAA, 1×10^−4^ M 2-Mecaptoenthanol (Invitrogen), 50 ug/ml Vitamin C (sigma), 0.5 mM sodium butyrate, 8 ng/ml recombinant human basic fibroblast growth factor (bFGF, PEPROTECH). iPSC and ES cells were maintained in Knockout DMEM containing 20% Knockout Serum Replacement (KSR, Invitrogen), 2 mM L-glutamine (Invitrogen), 1×10^−4^ M NEAA, 1×10^−4^ M 2-Mecaptoenthanol (Invitrogen), 20 ng/ml bFGF. The retroviral vectors expressing mouse miR-17∼92 cluster and deletion mutants were cloned in MSCV-PIG [33] (a gift from Andrea Ventura, Memorial Sloan Kettering Cancer Center). The PTEN shRNA in PLKO were purchased from Sigma. PTEN was PCR-amplified from human cDNA and cloned in pBABE vector. The primers are listed in Table S2.

### Human iPSC induction and characterization

The same retroviral vectors expressing Oct4, Sox2, Klf4 and cMyc were used to infect human fibroblastic IMR90 cells, following previously published protocols [34]. After retroviral transduction, treated IMR90 cells were re-plated on MEF feeders under the human ESC culture conditions. The derived human iPSC lines were characterized by standard methods [34].

### MicroRNA array

Total RNA of cultured IMR90 cells transfected with different combination of modified mRNAs coding for Yamanaka factors was submitted to the Johns Hopkins Microarray Core Facility. The modified mRNAs were synthesized by Integrated DNA Technologies (Coralville, IA). MicroRNA array analysis was performed using an Affymetrix platform as described in http://www.microarray.jhmi.edu/.

### Quantitative RT-PCR and Western blotting

For gene expression or silencing analysis, total RNA was isolated from cells using TRIzol (Invitrogen). First-strand cDNA was generated using iSCRIPT (Bio-rad), and real-time PCR was performed in triplicate using a SYBR Green PCR master mix. All values were normalized to the level of 18S rRNA, which are constitutively expressed and were unchanged during the experiments. The primer sequences used in the experiments are included in Table S2.

For miRNA analysis, total RNA was extracted using a mirVana kit (Invitrogen), and reverse-transcribed using specific stem-loop primers. 50 ng of small RNAs was used for SYBR Green qRT-PCR, and samples were analyzed in triplicate. U6 small nucleolar RNA was used as loading control. miRNA mimic and antagomir (RiboBio) were transfected on day 0, 3, 5 after infection using Lipofetamine 2000 (Invitrogen) following the instructions of the manufacturer. The primer sequences used in the experiments are included in Table S2.

Western blotting was performed using an ECL kit (Thermo) based on the manufacturer's recommendations. HRP-conjugated secondary antibodies were purchased from the Jackson Laboratory. Dilutions of primary antibodies were as follows: anti-PTEN (1∶1000, Cell Signaling Technology), anti-GAPDH (1∶5000, Abmart).

### Immuno-staining of undifferentiated and differentiated human iPS cells

For in-situ immuno-staining, cultured hESCs and iPS cells were fixed with 4% para-formaldehyde in PBS for 20 min and washed with PBS for 15 min. For embryoid body (EB) formation assays, EBs of 8 days were transferred onto gelatin-coated plates for additional 2-day attachment (after mild pipetting to break the EBs into smaller pieces). The fixed samples were incubated with the following primary antibodies for 2 hours at room temperature: anti-TRA-1-60 (Millipore), anti-SOX1 (Millipore), anti-OCT-4 (Millipore), anti-SSEA-4 (Millipore), anti-αSmooth Muscle Actin (Sigma), anti-Tubulin beta III isoform (Millipore), anti-AFP (Sigma). After wash with PBS, Alexa-488 or Texas Red conjugated goat anti-rabbit or anti-mouse secondary antibodies (1∶500, Invitrogen) were used for 1-hour incubation to visualize the cells together with DAPI nuclear staining.

### Teratoma formation

Typically cells at 80–90% confluence in two 10 cm dishes were used for the following procedure. Cells were collected into a 50 ml tube by directly scraping them in their native media using a cell-scraper. After spinning down, the cell pellet was resuspended on ice in 400 µl of a 1∶1 mixture of Matrigel (BD Biosciences) and knockout DMEM and collected in an eppendorf tube and stored on ice. This volume of suspension is suitable for intra-muscular injection into the hind limb of two SCID-beige mice (200 µl each). Palpable tumors were detected in 4–6 weeks post injection and tumors were allowed to grow for additional 2–3 months before sacrificing the animals. After sectioning, slides containing various regions of tumors were stained with H & E. Complex structures with various cell types were examined at both low and high magnitude.

### Karyotyping

Karyotype analysis was conducted using standard chromosome analysis protocols. Briefly, the iPS cells were cultured with daily medium change and were treated with colcemed solution before collection. After hypotonic treatment, cell suspension was fixed, prepared to make slide and stained with Giemsa solution.

### Statistical analyses

Data plotted are typically expressed as mean standard error of mean (SEM) unless otherwise indicated.

## Results

### cMyc regulates many miRNAs during early stage of reprogramming

**Figure 1:**
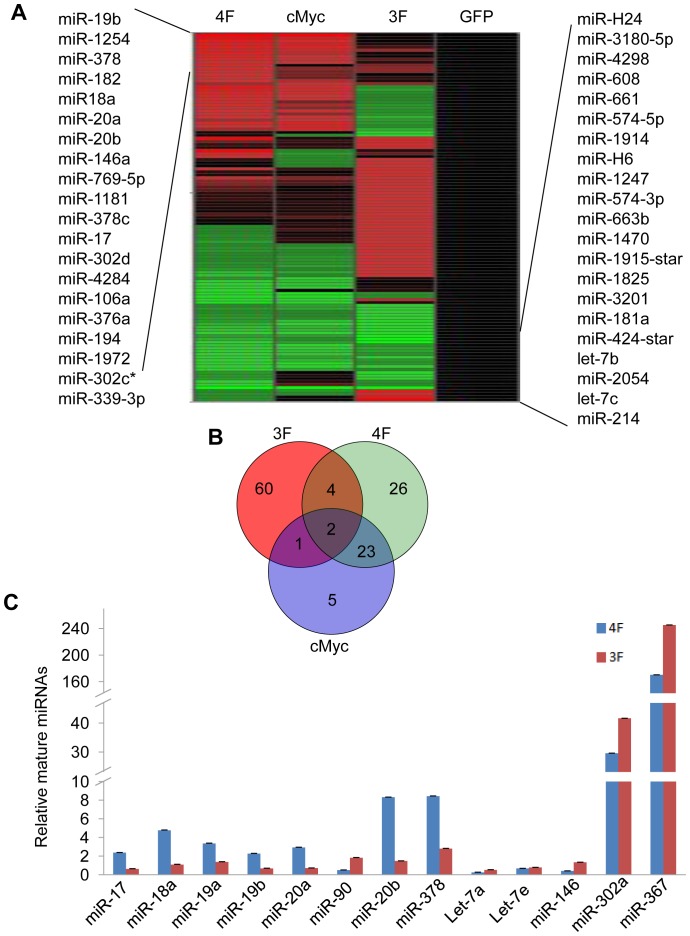
cMyc regulates many miRNAs during early stage of reprogramming. A. Heat map showing changes of 1174 human miRNAs in cMyc, 3F, or 4F transfected human fibroblast cells IMR90 as compared with GFP expressing vector -transfected cells. And120 miRNAs with more than 2-folds changes compared with GFP control group. B. Venn diagram showing numbers of miRNAs the expression of which changed more than 2-folds as compared with GFP control group. C. qRT-PCR analyze mature miRNA expression in IMR90 cell at day 3 after infected with viruses expressing Oct4, Sox2, and Klf4 with or without cMyc. The expression levels were normalized to that of the IMR90 infected with GFP control virus, U6 was used as the internal control. *Error bars, s.d.; n = 3*.

While cMyc was documented to modulate expression of numerous coding genes, its effect on miRNA expression in human somatic reprogramming was not clear. To investigate the role of cMyc targeted miRNAs in iPSC induction, we transfected human fibroblast cell line IMR90 with synthesized message RNA (mRNA) for OCT4, SOX2, KLF4, c-MYC, and GFP as control. The cells were transfected with mRNAs for 4F (OSKM), 3F (OSK), cMyc only, or GFP respectively as previously described [35]. After 72 hours, GFP expression demonstrated high transfection efficiency of this method (Figure. S1A). A small portion of cells were also stained for cMyc, Oct4 and Sox2 protein expression, which confirmed high expression of those proteins after transfection (Figure. S1A ). The remaining majority of cells were collected and total RNA were purified. The samples were then analyzed by the use of miRNA arrays. Of the 1174 human miRNAs tested, 121 miRNAs were induced with more than 2-folds changes in 4F, 3F, and cMyc only groups, as compared with GFP control group (Figure. 1A, and table S1). It is interesting to note that, while cMyc only group displayed substantial overlap with 4F group in terms of miRNAs induction, there is little overlap between 4F and 3F groups, or between 3F and cMyc only groups. Only 6 miRNAs were regulated by more than 2-fold changes in both 3F and 4F groups, while 67 members induced in 3F and 55 in 4F (Figure 1B). These results demonstrated that cMyc induced a distinct change of miRNA expression profile during early stage of human somatic reprogramming.

Our array data showed that four members of miR-17∼92 cluster were among the most significantly upregulated miRNAs in both 4F and cMyc only transfected cells (Figure 1A). We further validated the expression profile of miR-17∼92 by Real-time PCR in IMR90 infected with retrovirus encoding 4F, 3F or GFP as control. And the results showed that miRNAs of the miR-17∼92 cluster were indeed up-regulated by cMyc (Figure 1C). Of note, although the miRNA array did not detect miR-19a induction by cMyc, our repeated PCR assay did confirm that cMyc enhanced miR-19a expression. We also found that miR-302∼367 cluster were up-regulated both in 4F and 3F groups, but not in the cMyc only group, indicating that miR-302∼367 were not regulated by cMyc directly. Let-7s were down-regulated in 4F group compared with 3F. These results indicated that forced cMyc expression regulates many miRNAs in the early stage of human iPSCs induction, with miR-17∼92 cluster miRNAs being among the most significantly upregulated.

### miR-17∼92 cluster enhances human somatic cell reprogramming

**Figure 2:**
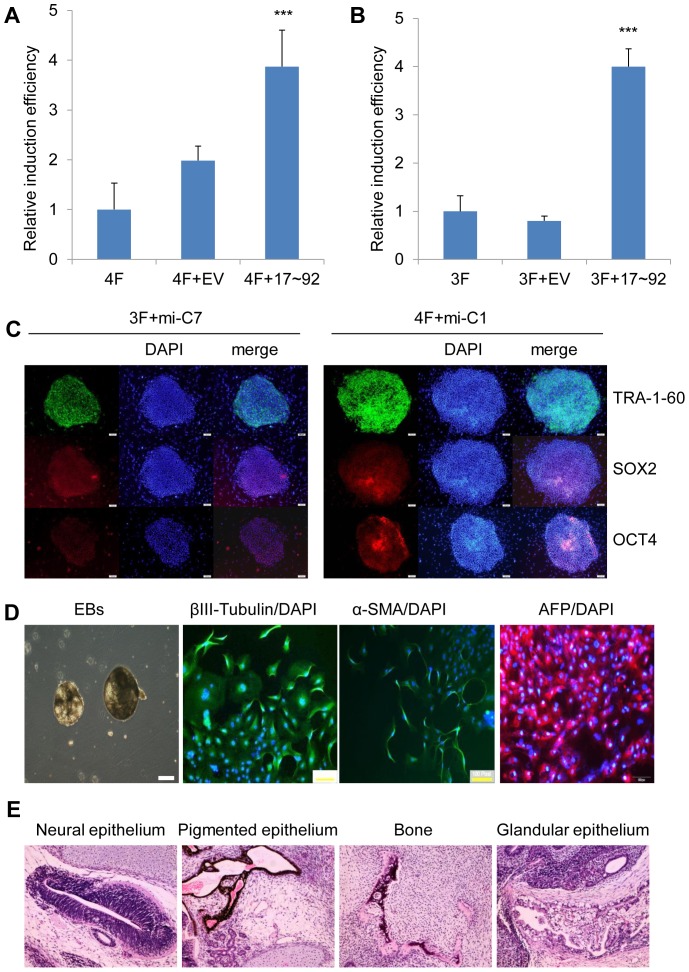
miR-17∼92 cluster enhances human somatic cell reprogramming. A. The relative induction efficiency normalized to that of 4F-induced reprogramming: IMR90 cells were infected with retroviruses containing 4F (Oct4, Sox2, Klf4, cMyc) in the presence of an additional retroviral vector expressing miR-17∼92 cluster or its empty vector, respectively. Stem cell morphology colonies were observed on day 10 and counted on day 18 after infection. The induction efficiency was normalized to that of 4F group, which was set as “1”. Error bars, s.d.; n = 3. *, p<0.05;**, p<0.01; ***, p<0.001. B. The relative induction efficiency normalized to that of 3F-induced reprogramming:IMR90 cells were infected with retroviruses containing 3F (Oct4, Sox2, Klf4) in the presence of an additional retroviral vector expressing miR-17∼92 cluster or its empty vector. Stem cell morphology colonies were counted on day 35 after infection. The induction efficiency was normalized to that of 3F, which was set as “1”. Error bars, s.d.; n = 3. *, p<0.05;**, p<0.01; ***, p<0.001. C. Immunofluorescence staining of ESC pluripotency markers TRA-1-81, TRA-1-60, OCT4 and SOX2 in iPSC colonies generated from IMR90 by 4F plus miR-17∼92 (4F+mi-C1) and 3F plus miR-17∼92 (3F-mi-C7) Nuclei were stained with DAPI (blue). Scale bars, 100 µm. D. iPSCs by 3F plus miR-17∼92 (3F+mi-C7) were culture in suspension condition to form embryoid bodies (left). Immunofluorescence staining showed that differentiation markers α-SMA (mesoderm), AFP (endoderm), βIII-tubulin (ectoderm) were all expressed in those formed EBs. Nuclei were stained with DAPI (blue). Scale bars, 100 µm. E. Teratoma assay showed that miR-17∼92 cluster derived iPSCs (4F+miR-C1) can be differentiated in vivo. The images magnified 100 times.

Having observed the significant induction of miR-17∼92 cluster miRNAs in the early stage of reprogramming process, we sought to further study the function of miR-17∼92 in the reprogramming. Using the pMSCV (retro-base)-PIG-17∼92 (PIG-17∼92) that encodes whole cluster of miR-17∼92 [27], we observed that HEK293T cells transfected with PIG-17∼92 can generate ∼3-fold of more mature miRNAs than control group (Figure S2A). To investigate the effect of miR-17∼92 on iPSC induction, we infected IMR90 cells with miR-17∼92 cluster, together with the four retroviral vectors expressing Oct4, Sox2, Klf4 and cMyc (OSKM, or 4F). After infection, the cells were cultured under standard condition for human iPSC induction, and monitored daily for morphological changes. The numbers of iPSCs clones were significantly increased by miR-17∼92 cluster as compared to 4F alone or 4F with control virus expression (Figure 2A). We next studied whether miR-17∼92 would increase the iPSC induction without cMyc (OSK, or 3F). For this purpose, we induced iPSCs using 3F combined with miR-17∼92 cluster or control, and found that, as with 4F, miR-17∼92 also significantly enhanced somatic reprogramming without cMyc (Figure 2B). These results showed that miR-17∼92 cluster promoted iPSC induction in the presence or absence of cMyc.

### The pluripotency and developmental potential of miR-17∼92-derived iPSCs

To determine whether miR-17∼92-enhanced reprogramming could generate *bona fide* iPSCs, we derived stable cell lines from cultures of 4F plus miR-17∼92 (4F+mi-C1 and 4F+mi-C3) and 3F plus miR-17∼92 (3F+mi-C7). The iPSCs generated with miR-17∼92 exhibited hESC morphology and were alkaline phosphatase (AP) and TRA-1-81 positive (Figure S2B). The iPSC clones 4F+mi-C1 and 3F+mi-C7 expressed pluripotent markers, TRA-1-60, OCT4 and SOX2, as detected by immunostaining (Figure 2C). RT-PCR analysis of mRNAs derived from iPSCs cell lines showed that the expression levels of pluripotency markers (OCT4, NANOG, SOX2 and REX) in these iPSCs cell lines were as high as in hESC cell line H9 (Figure S2C). Moreover, exogenous transgenes were silenced in all the iPSC cell lines compared with IMR90 cells infected with 4F for 6 days as the positive control (Figure S2D). These results suggest that the iPSCs induced by transduction with 4F and miR-17∼92 are pluripotent.

To examine the developmental potentials of the miR-17∼92-derived iPSCs, we used standard human embryoid bodies (EBs) cultivation assay (Figure 2D). After 8 days of suspension culture, cystic forms of EBs were formed. Next we transferred the cells to gelatin-coated plates and continued to culture for another 8 days. Immunostaining showed that the derived iPSCs 3F+mi-C7 differentiated successfully into different cells expressing α-SMA (mesoderm), AFP (endoderm), orβIII-tubulin (ectoderm) (Figure 2D). Further, iPSCs showed pluripotency *in vivo* by teratoma formation assay (Figure 2E). Karyotyping analysis confirmed that those iPSCs (4F+mi-C1 and 3F+mi-C7) had normal karyotypes (Figure S2E).

### miR-19a and miR-19b are the key components of miR-17∼92 cluster in human fibroblast reprogramming

**Figure 3:**
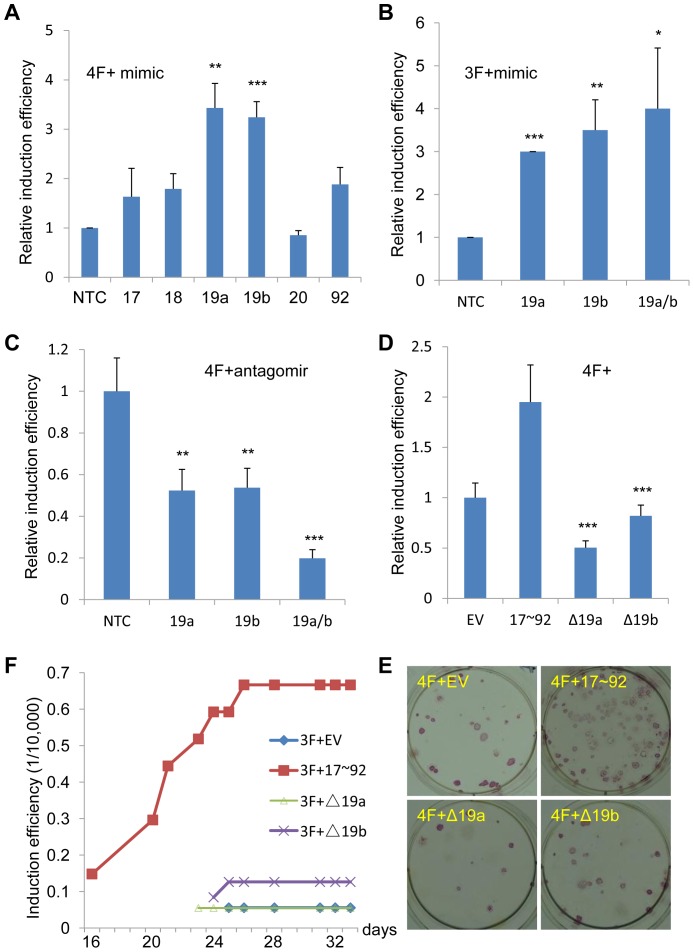
miR-19a and miR-19b are the key components of miR-17∼92 cluster in reprogramming. A. 4F-infected IMR90 cells were further transfected with miRNA mimics or control oligo three times (on day 0, 3 and 5 after 4F infection, respectively). The numbers of formed iPSCs were counted on the 18^th^ day and normalized to that of 4F plus non-target control (NTC) oligo group. Error bars, s.d.; n = 3. **, p<0.01; ***, p<0.001. B. miR-19a and/or miR-19b stimulated the reprogramming in IMR90 cells induced by 3F. The iPSC induction efficiency was normalized to 3F plus NTC. Error bars, s.d.; n = 3. *, p<0.05;**, p<0.01; ***, p<0.001. C. Inhibition of miR-19a and/or miR-19b decreased the reprogramming in IMR90 cells induced by 4F. The induction efficiency was normalized to 4F plus NTC ologo group. Error bars, s.d.; n = 3. *, p<0.05;**, p<0.01; ***, p<0.001. D. Deletion of miR-19a or miR-19b in miR-17∼92 cluster decreased the efficiency of reprogramming in IMR90 cells induced by 4F. The induction efficiency was normalized to 4F plus empty vector (EV) group. Error bars, s.d.; n = 3. *, p<0.05;**, p<0.01; ***, p<0.001. E. A representative image of alkaline phosphatase staining for figure 3D was showed. F. Deletion of miR-19a or miR-19b in miR-17∼92 cluster decreased the efficiency of reprogramming in IMR90 cells induced by 3F. The induction efficiency was calculated as 1/10000.

The miR-17∼92 cluster is a primary transcript that processes six mature miRNAs: miR-17, miR-18a, miR-19a, miR-20a, miR-19b and miR-92a. To determine the key components of miR-17∼92 that facilitate reprogramming, we used mimics of these six miRNAs in 4F induction. First, we examined the overexpression efficiency of miRNA mimics (Figure S3A). Then we used these miRNA mimics in the iPSC induction experiments. We found that, among those miRNA mimics, only miRNA mimics for miR-19a and miR-19b significantly enhanced iPSC induction efficiency by 4F (Figure 3A). Although other miRNAs such as miR-18a and miR-92 also increased the iPSC induction efficiency, their effect was marginal. Further, we induced iPSCs with miR-19a and/or miR-19b mimics combined with 3F, or miR-19a and/or miR-19b antagomirs combined with 4F. We found that iPSC induction efficiency by 3F was also greatly enhanced in the presence of miR-19a and/or miR-19b mimics (Figure 3B). Moreover, iPSC induction efficiency by 4F decreased significantly after inhibition of miR-19a or/and miR-19b by antagomirs (Figure 3C). The inhibitory efficiency of miRNA antagomirs was confirmed by real-time PCR (Figure S3B). To further validate the results of the antagomir experiment, we used miR-19a- or miR-19b- truncated miR-17∼92 cluster expressing vector for iPSC induction [33]. Real-time PCR analysis showed that truncation of miR-19a and miR-19b specifically reduced mature miRNA expression of miR-19a and miR-19b, respectively, without affecting the maturation of other miRNAs in this cluster (Figure S3C). Not like the whole cluster, the truncated forms of miR-17∼92 failed to enhance the number of 4F-induced iPSCs (Figure 3D, 3E). In case of iPSC induction by 3F, deletion of miR-19a or miR-19b not only resulted in decreased iPSC clone numbers but also delayed the appearance of iPSC clones (Figure 3F). Taken together, the data indicate that miR-19a and miR-19b are the key components of miR-17∼92 cluster in human fibroblastic cell reprogramming.

### PTEN is a target of miR-19a/b in human fibroblast reprogramming

**Figure 4:**
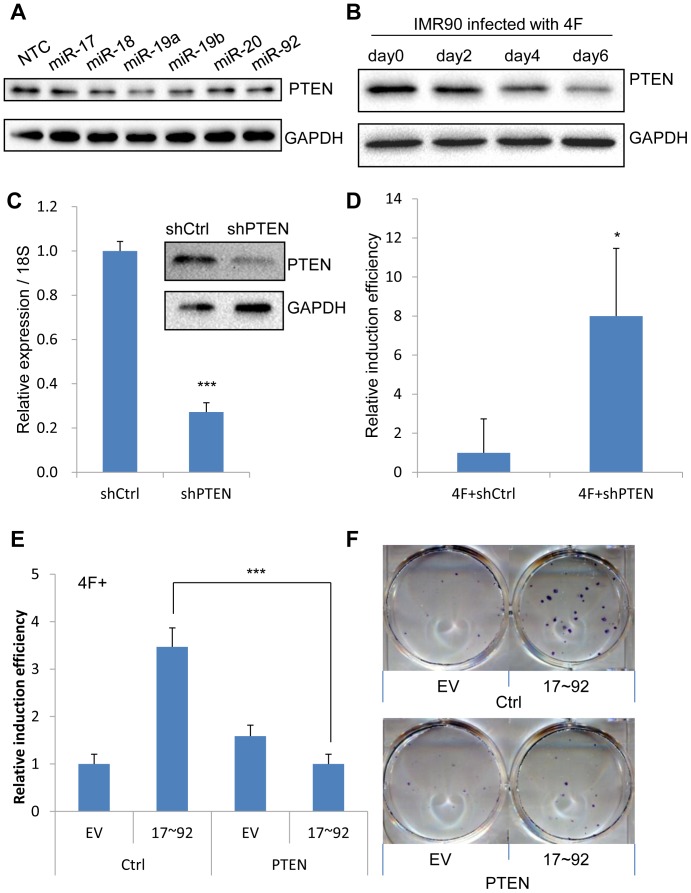
PTEN is a target of miR-19a/b that suppresses reprogramming. A. Western blot analysis showed that PTEN protein expression decreased in IMR90 cells transfected with miR-19a or miR-19b mimics. GAPDH was used as loading control. B. Western blot analysis showed that PTEN protein expression decreased gradually during the early stage of iPS induction in IMR90 cells by 4F. GAPDH was used as loading control. C. qRT-PCR and western blot analysis confirmed the knockdown of PTEN with its shRNAs in IMR90 cells. 18S and GAPDH were used as loading control in qRT-PCR and western blot, respectively. *Error bars, s.d.; n = 3*. *, *p<0.05*;**, p<0.01; ***, p<0.001. D. Knockdown of PTEN enhanced the reprogramming of IMR90 cells induced by 4F. The induction efficiency was normalized to 4F plus shRNA control (shCtrl). *Error bars, s.d.; n = 3*. *, *p<0.05*;**, p<0.01; ***, p<0.001. E. The enhancing effect of miR-17∼92 on iPSC induction with IMR90 cells was attenuated by PTEN overexpression. Error bars, s.d.; n = 6. *, p<0.05;**, p<0.01; ***, p<0.001. F. A representative image of alkaline phosphatase staining for figure 4E was showed.

It is previously reported that miR-19a/b suppress PTEN by directly targeting its 3′UTR in cancer cells [36]. To determine whether miR-19a/b could suppress PTEN in human somatic cells during reprogramming, we detected PTEN levels in IMR90 cells transfected with miRNA mimics. Western blot showed that PTEN protein levels decreased in cells transfected with miR-19a or miR-19b compared with that of NTC control group (Figure 4A), while the other miRNA mimics did not significantly affect PTEN protein levels. We also observed a gradual decrease in PTEN protein expression during reprogramming progress induced by 4F (Figure 4B). However, the expression of PTEN was not decreased during fibroblast reprogramming without cMyc (Figure S4C). To directly examine the effect of PTEN on iPSC induction, we next manipulated PTEN during reprogramming of IMR90 cells by gain of function and loss of function assays. The efficiency of PTEN overexpression was determined by western blotting analysis (Figure S4A). However, iPSC generation was not significantly affected when PTEN was overexpressed (Figure S4B). Then, we knocked down PTEN in IMR90 by shRNA and the efficiency of knockdown was confirmed by western blotting and qRT-PCR analysis (Figure 4C). We found that the efficiency of iPSCs induction by 4F or 3F was increased dramatically with knocking-down of PTEN (Figure 4D, Figure S4D). To further determine whether miR-17∼92 enhanced reprogramming by repressing PTEN, we combined PTEN overexpression with miR-17∼92 in the 4F-induced reprogramming. To this end, we induced iPSCs in the presence of 4F together with miR-17∼92 and PTEN that didn't contain the targeting sequence of miR-19a/b. Compared to the combination of miR-17∼92 with EV control, we found that overexpression of PTEN lacking the targeting sequence of miR-19a/b abrogated the stimulating effects of miR-17∼92 on reprogramming (Figure 4E, 4F). Collectively, these results indicate that tumor suppressor PTEN is a major target of miR-17∼92 in facilitating iPSC induction of human fibroblasts.

## Discussion

While iPSCs generated by somatic reprogramming with transcription factors hold great promise in regenerative medicines for cell therapy, they also pose many problems such as induction efficiency and safety concerns. Therefore, it is critical to decipher how an individual factor functions during the reprogramming process. In this study, we focused on the role of cMyc-mediated miRNAs in reprogramming of human fibroblasts. By microarray and qRT-PCR analysis, we discovered that cMyc regulated many miRNAs, most notably miR-17∼92 cluster members, during early stage of reprogramming. We found that miR-17∼92 cluster, miR19a and miR19b in particular, enhanced human fibroblast reprogramming, in the presence or absence of cMyc. Importantly, we further demonstrated that the enhancement of reprogramming by miR-17∼92 was mediated by suppression of tumor suppresser protein PTEN. Taken together, our findings in this study represent the first demonstration that cMyc-miR-19a/b-PTEN axis plays a pivotal role in reprogramming of human somatic cells.

*cMyc* is a proto oncogene that plays a pivotal role in cell growth, proliferation, tumorigenesis, and biomass accumulation [12]. *cMyc* is previously reported to regulate miRNAs in cancer cells and their mechanisms in tumorigenesis are extensively studied [37]. However, it is unknown which miRNAs are induced by *cMyc* and what roles they might play during human somatic cell reprogramming. Our results showied that four out of the six miR-17∼92 cluster miRNAs were significantly induced by cMyc during early stage of reprogramming suggested that those miRNAs might be critical for human somatic reprogramming. Supporting our assumption was a recent report that members of this cluster miRNAs could stimulate reprogramming in a mouse system [38]. However, it remained unclear if human reprogramming would behave the same as in the mouse system. It was also unknown as to which miRNA(s) of this cluster would be more responsible and what would be their target genes. Hence, we focused to decipher the roles miR-17∼92 cluster miRNAs might play during reprogramming of human fibroblast cells and its underlying mechanisms. Our studies demonstrate for the first time that miR-17∼92 cluster stimulates human fibroblast reprogramming by targeting PTEN, with miR-19a and miR-19b playing a predominant role. Data leading to this concludion include the following: Firstly, we provided new evidence to show that *cMyc could* stimulate expression of miR-17∼92 cluster miRNAs, among others, during early stage of human fibroblastic cell reprogramming; Secondly, forced expression of miR-17∼92 cluster with 4F or 3F enhanced human iPSC induction; Thirdly, miR-19a and miR-19b of the miR-17∼92 cluster were key members that play critical roles in this process; Lastly, PTEN was a target of miR-19a/b that mediated the effect of miR-17∼92 cluster on human fibroblast reprogramming.

The revelation of the key roles of cMyc-miR-19a/b-PTEN axis in reprogramming of human fibroblast cells is very interesting. Many proto-oncogenes such as *cMyc, Sox2, Ras, lin28, Akt* and *SV40* are stimulators of reprogramming, while tumor suppressor genes like *P53* are the barriers. Here, added to the list are oncomirs mir-19a/b and tumor suppressor PTEN, suggesting important nodals existing at the crossroad of reprogramming and oncogenesis. Not surprisingly, previous studies have demonstrated that iPS cells generated by transduction with defined transcription factors displayed high potential of oncogenesis [32]. *Okita et al* reported that the reactivation of cMyc in iPSCs led to tumor formation. *Tong et al* reported that all iPS mice were prone to develop tumors [39]. The significance of these observations was obscured by the fact that iPSCs were successfully induced without cMyc [14]. However, those results are too important to be simply ignored because further insights might provide the key to understanding the fate decision and safety control during reprogramming of human somatic cells. While the detailed mechanisms remain to be elucidated, results of the present study seem to suggest the assumption that, since reprogramming signaling shares substantially with tumorigenic cues, the cells with tumorigenic potentials are more easily to be induced to pluripotency, and vice versa. Supporting this assumption is our recent demonstration that Eras/Akt/Foxo1 axis facilitates reprogramming in mouse and human models^29^. Hence, we surmise that it is not by a mere coincidence to discover that, mir19a/b, known to be the most oncogenic miRNAs of miR-17∼92 cluster in tumorigenesis progress [27], [36], are also key components to facilitate reprogramming of human somatic cells. While little is known about the long-term oncogenic potentials of various iPSC cells generated by different methods, it is relevant to study the activation status of tumorigenic signaling in all iPS cells so as to work out safe measures for their eventual applications in regenerative medicines.

Of note, other cMyc-mediated miRNAs and targets might also be involved in reprogramming. For instance, our data showed that miRNA let-7, famously known as associated with stem cell functions, and a less known miR-150, were significantly down-regulated by cMyc [40], suggesting that, as in cancer cells, cMyc's roles in reprogramming are multifaceted and complex mechanisms are likely involved. Hence, while our current results clearly establish cMyc/miR19/PTEN axis as key players in this process, further deciphering these roles and underlying mechanisms will no doubt further our understanding of the fate decision and safety controls during reprogramming.

## Supporting Information

Figure S1 **The high transfection efficiency of modified mRNAs.** A. IMR90 cells transfected with modified mRNAs for cMyc, Klf4, Oct4, Sox2 and GFP for 72 hours. GFP expression was observed under microscope. The expression of cMyc, Oct4 and Sox2 was determined by immunofluorescence staining.
(TIF)

Figure S2 **The iPSCs clones derived from miR-17∼92 are** ***bona fide*** **iPS cells.** A. qRT-PCR analysis showed that the expression of mature miRNAs were increased in 293T cells transiently transfected with vector of PIG-17∼92 cluster. The expression levels were normalized to that of 293T transfected with NTC. U6 was used as internal control. Error bars, s.d.; n = 3. *, p<0.05;**, p<0.01; ***, p<0.001. B. The induced human iPSCs we generated (4F+mi-C1) showed normal morphology (top left), AP positive (bottom left) and TRA-1-81 positive (top right). *Scale bars, 100 µm*. C. Reverse-transcript PCR analysis of the pluripotency genes in the iPSC clones generated from IMR90 cells induced by 3F or 4F in the presence or absence of miR-17∼92 as indicated. IMR90 cells were used as negative control, and human H9 ES cells were used as positive control. 18S was used as loading control. D. Reverse-transcript PCR analysis of exogenous genes in the iPSCs we generated and IMR90 transfected with 4F for 6 days using p-MX vector primers showed that exogenous were silenced after several passages of standard hESCs cultivation. IMR90 cells and H9 cells were used as negative control, and IMR90 cells infected with 4F for 6 days were used as a positive control. 18S was used as loading control. E. The iPSC cells 4F+mi-C1 and 3F+mi-C7 showed 44 normal chromosomes and two X chromosomes, a normal female karyotype.
(TIF)

Figure S3 **The mature miRNA expression efficiency of miRNA mimics, antagomirs, and the truncated form of PIG-17∼92.** A. Mature miRNA Expressions of miR-17-92 cluster were analyzed by qRT-PCR in 293T cells transfected with different miRNA mimics. The expression levels were normalized to cells transfected with non-target control (NTC). Error bars, s.d.; n = 3. *, p<0.05; **, p<0.01; ***, p<0.001. B. Mature miR-19a and miR-19b expressions were analyzed by qRT-PCR in 293T cells transfected with miRNA antagomirs for miR-19a and miR-19b, respectively. The expression levels were normalized to cells transfected with non-target control (NTC) antagomir group. *Error bars, s.d.; n = 3*. C. Mature miRNA expressions of miR-17-92 cluster were analyzed by qRT-PCR in 293T cells transfected with miR-19a or miR-19b truncated vector or vector expressing the complete miR-17∼92 cluster. The expression levels were normalized to cells transfected with empty vector (EV) group. *Error bars, s.d.; n = 3*.
(TIF)

Figure S4 **Over expression of PTEN does not affect iPSC generation.** A. PTEN protein expression was analyzed by western blot in IMR90 cells infected with control virus or virus expressing PTEN. GAPDH was used as loading control. B. IMR90 cells were infected with control virus (EV) or virus expressing PTEN in the presence of 4F. 18 days after infection, the formed iPSC clones were counted, and the induction efficiency was normalized to that of 4F plus empty vector (EV) group. *Error bars, s.d.; n = 3. NS: p = 0.746*. C. Western blot analysis showed that PTEN protein expression was not changed during the early stage of iPS induction in IMR90 cells by 3F. GAPDH was used as loading control. D. Knockdown of PTEN enhanced the reprogramming of IMR90 cells induced by 3F. The induction efficiency was normalized to 3F plus shRNA control (shCtrl). *Error bars, s.d.; n = 2*.
(TIF)

Table S1 **The simplified array data showed miRNA expression in the early stage of human somatic reprogramming.**
(PDF)

Table S2 **All the primers we used.**
(PDF)
