# Supplementary figures and images for: Human Fibroblast Reprogramming to Pluripotent Stem Cells Regulated by the miR19a/b-PTEN Axis
Source: PLoS One. 2014 Apr 16;9(4):e95213. doi: 10.1371/journal.pone.0095213 (PMC3989277; doi:10.1371/journal.pone.0095213)

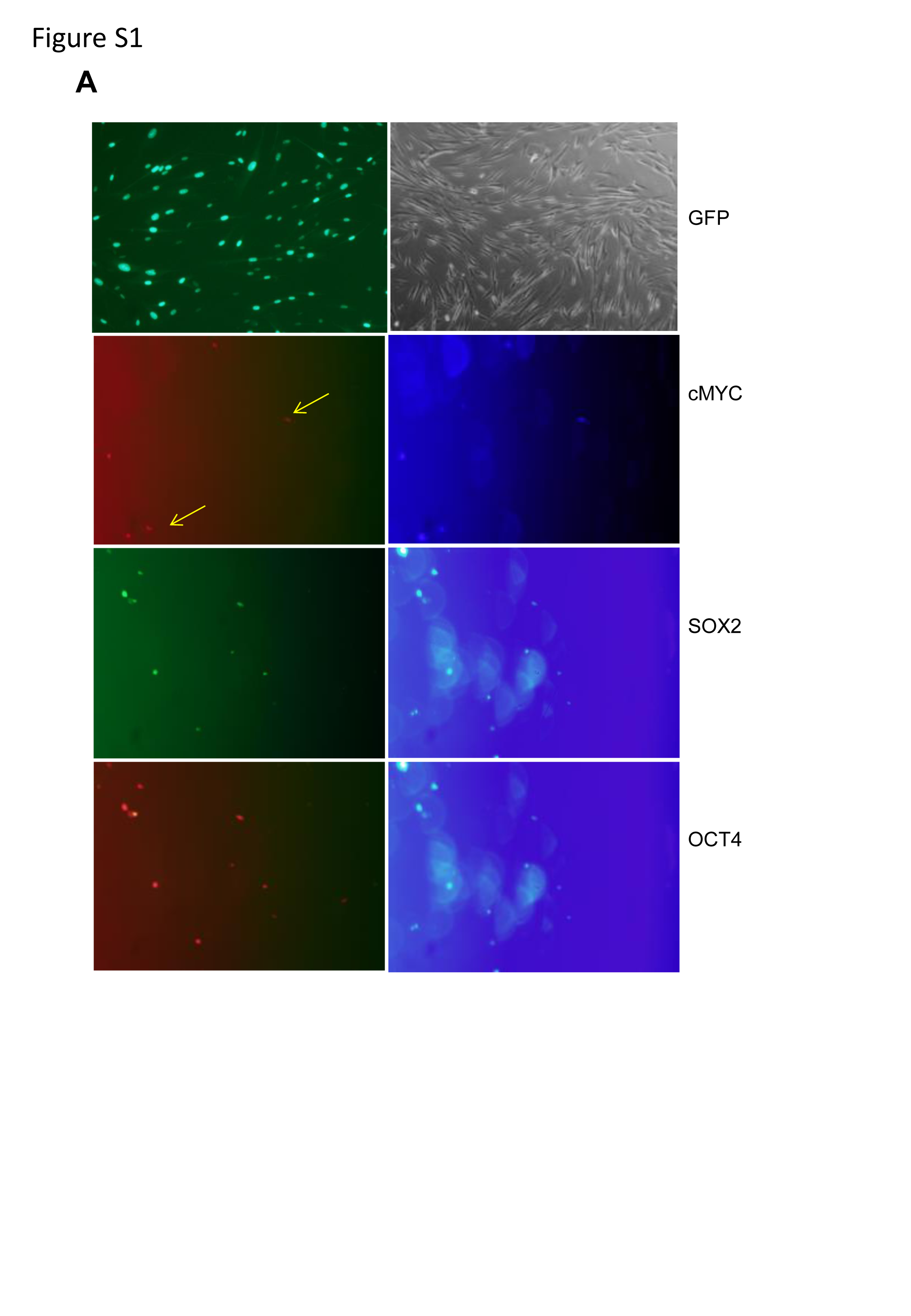

Supplement: Figure S1 — The high transfection efficiency of modified mRNAs. A. IMR90 cells transfected with modified mRNAs for cMyc, Klf4, Oct4, Sox2 and GFP for 72 hours. GFP expression was observed under microscope. The expression of cMyc, Oct4 and Sox2 was determined by immunofluorescence staining. (TIF) [file pone.0095213.s001.tif]

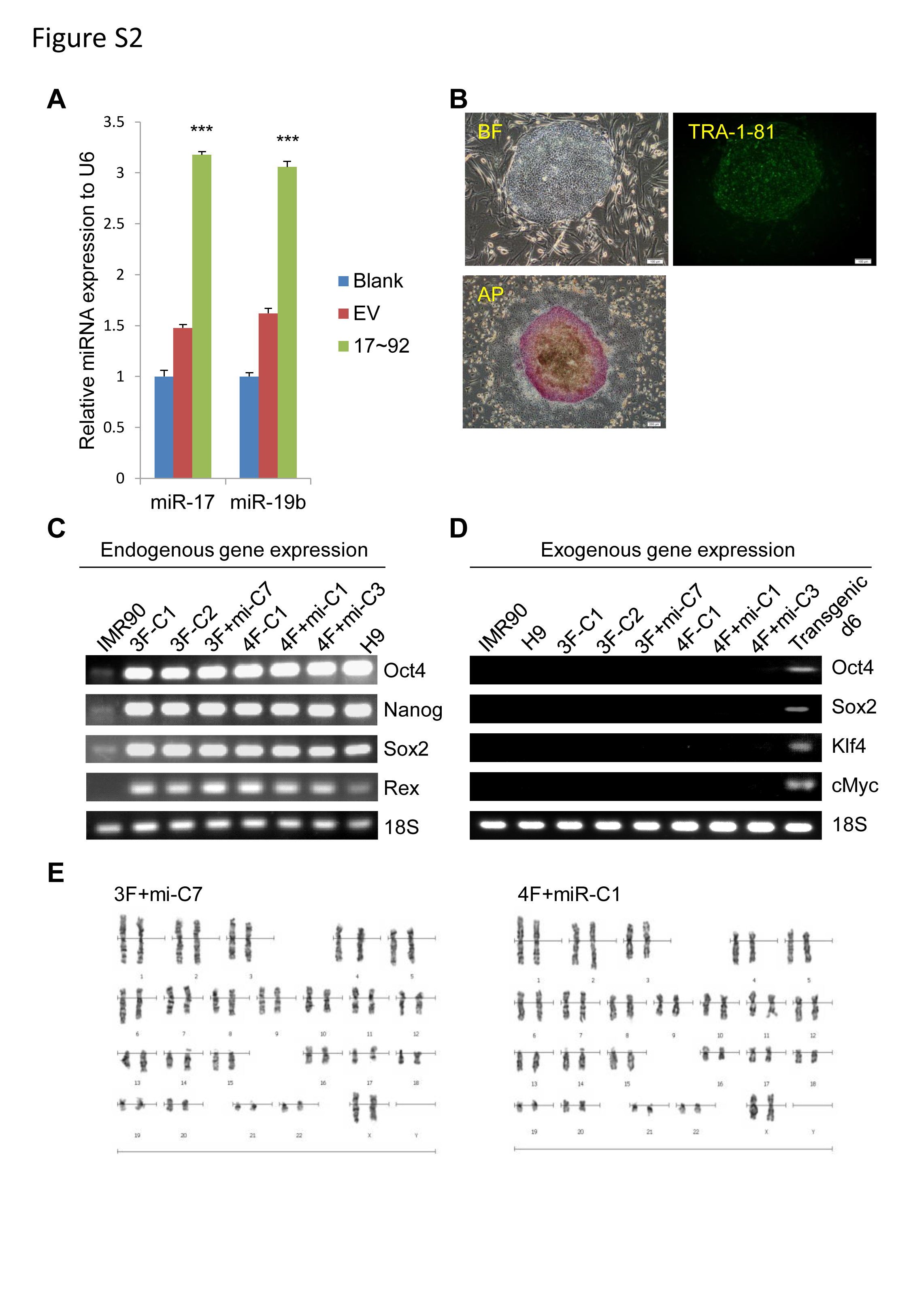

Supplement: Figure S2 — The iPSCs clones derived from miR-17∼92 are bona fide iPS cells. A. qRT-PCR analysis showed that the expression of mature miRNAs were increased in 293T cells transiently transfected with vector of PIG-17∼92 cluster. The expression levels were normalized to that of 293T transfected with NTC. U6 was used as internal control. Error bars, s.d.; n = 3. *, p<0.05;**, p<0.01; ***, p<0.001. B. The induced human iPSCs we generated (4F+mi-C1) showed normal morphology (top left), AP positive (bottom left) and TRA-1-81 positive (top right). Scale bars, 100 µm. C. Reverse-transcript PCR analysis of the pluripotency genes in the iPSC clones generated from IMR90 cells induced by 3F or 4F in the presence or absence of miR-17∼92 as indicated. IMR90 cells were used as negative control, and human H9 ES cells were used as positive control. 18S was used as loading control. D. Reverse-transcript PCR analysis of exogenous genes in the iPSCs we generated and IMR90 transfected with 4F for 6 days using p-MX vector primers showed that exogenous were silenced after several passages of standard hESCs cultivation. IMR90 cells and H9 cells were used as negative control, and IMR90 cells infected with 4F for 6 days were used as a positive control. 18S was used as loading control. E. The iPSC cells 4F+mi-C1 and 3F+mi-C7 showed 44 normal chromosomes and two X chromosomes, a normal female karyotype. (TIF) [file pone.0095213.s002.tif]

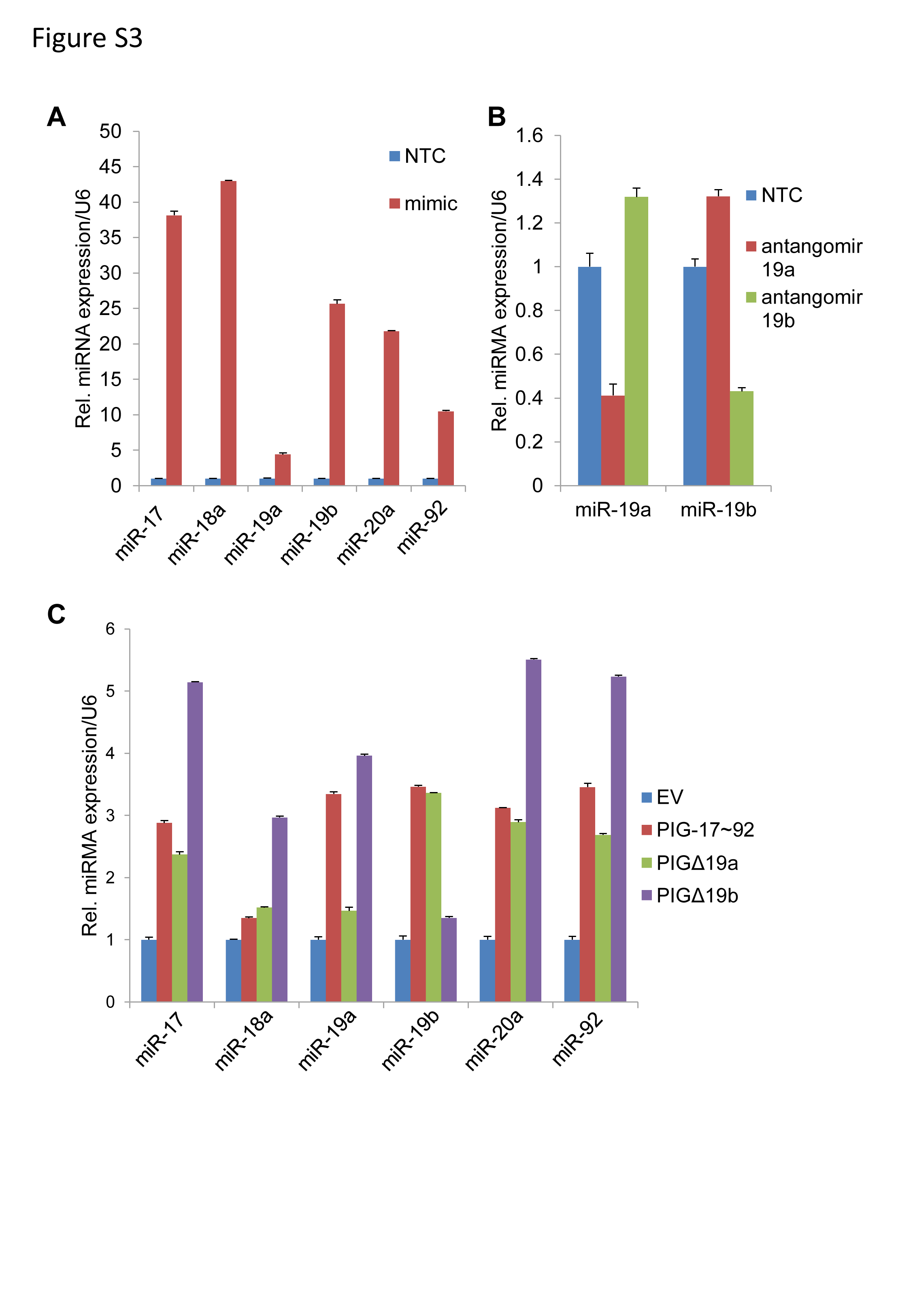

Supplement: Figure S3 — The mature miRNA expression efficiency of miRNA mimics, antagomirs, and the truncated form of PIG-17∼92. A. Mature miRNA Expressions of miR-17-92 cluster were analyzed by qRT-PCR in 293T cells transfected with different miRNA mimics. The expression levels were normalized to cells transfected with non-target control (NTC). Error bars, s.d.; n = 3. *, p<0.05; **, p<0.01; ***, p<0.001. B. Mature miR-19a and miR-19b expressions were analyzed by qRT-PCR in 293T cells transfected with miRNA antagomirs for miR-19a and miR-19b, respectively. The expression levels were normalized to cells transfected with non-target control (NTC) antagomir group. Error bars, s.d.; n = 3. C. Mature miRNA expressions of miR-17-92 cluster were analyzed by qRT-PCR in 293T cells transfected with miR-19a or miR-19b truncated vector or vector expressing the complete miR-17∼92 cluster. The expression levels were normalized to cells transfected with empty vector (EV) group. Error bars, s.d.; n = 3. (TIF) [file pone.0095213.s003.tif]

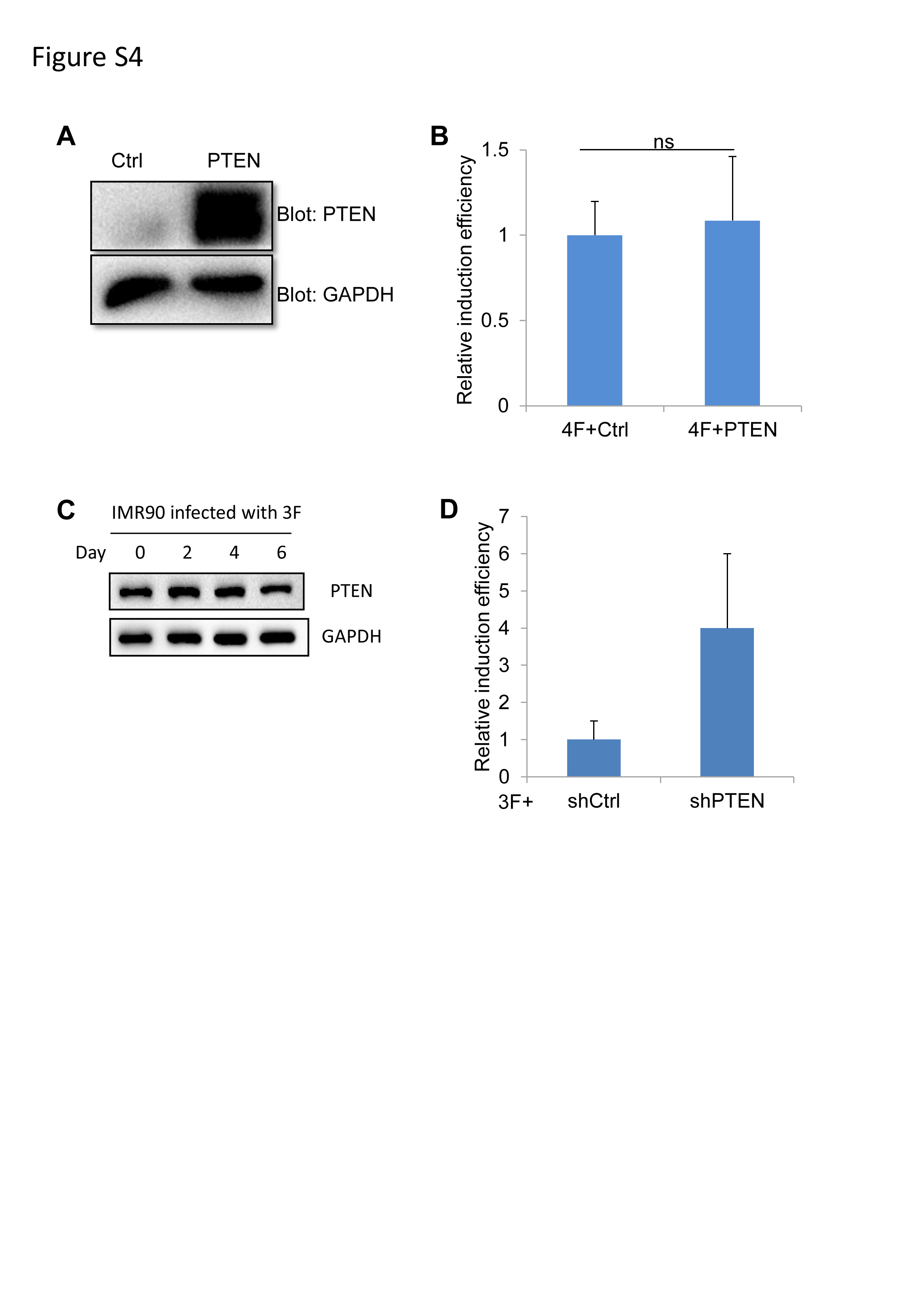

Supplement: Figure S4 — Over expression of PTEN does not affect iPSC generation. A. PTEN protein expression was analyzed by western blot in IMR90 cells infected with control virus or virus expressing PTEN. GAPDH was used as loading control. B. IMR90 cells were infected with control virus (EV) or virus expressing PTEN in the presence of 4F. 18 days after infection, the formed iPSC clones were counted, and the induction efficiency was normalized to that of 4F plus empty vector (EV) group. Error bars, s.d.; n = 3. NS: p = 0.746. C. Western blot analysis showed that PTEN protein expression was not changed during the early stage of iPS induction in IMR90 cells by 3F. GAPDH was used as loading control. D. Knockdown of PTEN enhanced the reprogramming of IMR90 cells induced by 3F. The induction efficiency was normalized to 3F plus shRNA control (shCtrl). Error bars, s.d.; n = 2. (TIF) [file pone.0095213.s004.tif]
