# Supplementary material for: Human Fibroblast Reprogramming to Pluripotent Stem Cells Regulated by the miR19a/b-PTEN Axis
Source: PLoS One. 2014 Apr 16;9(4):e95213. doi: 10.1371/journal.pone.0095213 (PMC3989277; doi:10.1371/journal.pone.0095213)
Supplement: Table S1 — The simplified array data showed miRNA expression in the early stage of human somatic reprogramming. (PDF) [file pone.0095213.s005.pdf]

Table S1

miRNA expression in the early stage of human somatic reprogramming

ProbeSet Name	Myc vs GFP Fold Change	3F vs GFP Fold Change	4F vs GFP Fold Change
hbv-miR-B20_st	0.082013791	-160.1800384	-140.077641
hbv-miR-B2RC_st	0.33164123	0.20962296	0.098020965
hbv-miR-B4_st	0.975475239	0.216034857	0.31312474
hcmv-miR-UL112_st	-180.9630633	0.04591249	-30.93135081
hcmv-miR-UL148D_st	0.000206626	-20.01258118	-4.421394099
hcmv-miR-UL22A_st	-211.8783225	0.063205228	0.043870978
hcmv-miR-UL22A-star_st	-335.0041262	-26.22285776	-5.79437208
hcmv-miR-UL36_st	0.005933544	0.027949988	6.25206E-05
hcmv-miR-UL36-star_st	-13.31303275	-24.80798818	-5.066978219
hcmv-miR-UL70-3p_st	0.872340122	0.048010069	0.657690993
hcmv-miR-UL70-5p_st	0.471081813	0.222476834	0.282451326
hcmv-miR-US25-1_st	0.068015075	0.042163284	-18.03590978
hcmv-miR-US25-1-star_st	-17.00085867	-12.04837727	-11.32652695
hcmv-miR-US25-2-3p_st	0.382550909	0.06890625	0.004061194
hcmv-miR-US25-2-5p_st	0.037776004	0.25849293	0.080963581
hcmv-miR-US33-3p_st	-14.44503799	0.074645343	0.0079512
hcmv-miR-US33-5p_st	0.0170009	0.038926304	0.0461152
hcmv-miR-US4_st	0.162513394	0.10846253	0.053210494
hcmv-miR-US5-1_st	-62.92571644	-498.9587171	0.002840464
hcmv-miR-US5-2_st	-96.77325021	-51.75641073	-17.19941666
hiv1-miR-H1_st	0.240422528	-1.219731778	0.131405888
hiv1-miR-N367_st	-247.9302116	0.059397489	0.006325578
hiv1-miR-TAR-3p_st	0.000747312	0.00221342	0.012796926
hiv1-miR-TAR-5p_st	0.002512917	0.001740725	0.041477803
hsa-let-7a_st	-17381.56312	-4351.125375	-27.78705014
hsa-let-7a-2-star_st	0.214574158	-27.11769613	-37.90620371
hsa-let-7a-star_st	0.180381132	0.064696975	0.06713566
hsa-let-7b_st	-1.160816739	-15.39076932	-0.773778376
hsa-let-7b-star_st	0.007742944	0.00628088	-83.64383122
hsa-let-7c_st	-1.320627413	0.001946221	-0.873845387
hsa-let-7c-star_st	0.136857083	0.151108292	0.43116427
hsa-let-7d_st	-6.357459905	-102.4231672	-8.758376621
hsa-let-7d-star_st	-22042.47308	-6.531092784	-190.84151
hsa-let-7e_st	-6.644802422	-1248.169832	-1200.624901
hsa-let-7e-star_st	-69.71548993	0.023347534	-40.00576002
hsa-let-7f_st	0.162955524	-8.367049669	0.06003284
hsa-let-7f-1-star_st	0.011657521	0.001361573	0.001967854
hsa-let-7f-2-star_st	0.267954275	0.086155164	0.06170107
hsa-let-7g_st	0.664589355	-51.93485201	0.685585656
hsa-let-7g-star_st	-52.38690935	0.000474303	0.060383724
hsa-let-7i_st	0.127708314	0.415355759	0.476142781
hsa-let-7i-star_st	-6.045844801	-5.480111066	-60.04691681
hsa-miR-1_st	0.000106761	-6.334870315	-71.65985345
hsa-miR-100_st	0.082748276	0.001213129	0.056019789
hsa-miR-100-star_st	-793.8734737	-24.60755664	0.037035848
hsa-miR-101_st	-2.960078363	-12.43890216	-3.974736306
hsa-miR-101-star_st	0.011412328	-37637848.62	0.087846439

hsa-miR-103_st	0.012446749	-660982.2196	0.31642875
hsa-miR-103-2-star_st	-200.2274756	-230.1713439	0.000109589
hsa-miR-103-as_st	-1231.148046	-68.37026347	0.000638623
hsa-miR-105_st	0.02571533	-629.729802	0.005183496
hsa-miR-105-star_st	0.029213988	-750.6304356	0.017946353
hsa-miR-106a_st	1.061605667	-14395.96885	2.182260176
hsa-miR-106a-star_st	-101.3484867	-123.5872089	-8.872596788
hsa-miR-106b_st	0.406406888	0.09245522	1.195637276
hsa-miR-106b-star_st	-1.318979276	-0.772503732	-6.257992032
hsa-miR-107_st	0.041257734	0.002755725	0.2652353
hsa-miR-10a_st	0.053378095	-5.353310529	-10680.91062
hsa-miR-10a-star_st	0.608021759	0.277912953	0.986191004
hsa-miR-10b_st	-1147.383098	-0.982183657	-98.15882103
hsa-miR-10b-star_st	-4430.846099	0.049360398	-55.09882013
hsa-miR-1178_st	-6.681782659	-8.496317187	-3.821381758
hsa-miR-1179_st	0.070555313	0.03146331	0.043092363
hsa-miR-1180_st	-1.598656661	-2.073366781	-3.521880161
hsa-miR-1181_st	2.92108092	0.695289146	2.769393558
hsa-miR-1182_st	0.136501061	9.327261105	0.457224802
hsa-miR-1183_st	-7.469035073	14.00464837	-55.39695182
hsa-miR-1184_st	-4.534438101	-0.922105687	-112.6048499
hsa-miR-1185_st	0.017331591	-8.761306319	0.009437151
hsa-miR-1193_st	0.065365871	0.004925583	0.149606117
hsa-miR-1197_st	0.004681502	-4.188584664	0.010511478
hsa-miR-1200_st	-12.22241679	-15.32540597	-13.02075151
hsa-miR-1201_st	0.008907962	-19.70191582	-1327.087264
hsa-miR-1202_st	0.049692212	-29.00838444	0.277763783
hsa-miR-1203_st	0.011095568	0.396624108	0.049763348
hsa-miR-1204_st	0.060581946	0.427190346	0.040976083
hsa-miR-1205_st	-140.0676942	0.035588634	0.013155402
hsa-miR-1206_st	0.105586978	0.113088946	0.095279021
hsa-miR-1207-3p_st	0.207370034	0.103409194	0.018799975
hsa-miR-1207-5p_st	-3.125761659	1.501030502	-1.514697821
hsa-miR-1208_st	0.345138625	-11.2372491	0.00945873
hsa-miR-122_st	0.279780697	0.674164977	0.163850896
hsa-miR-1224-3p_st	-2.162206835	-10.97547677	-3.660845616
hsa-miR-1224-5p_st	0.175117559	0.810221415	0.717079555
hsa-miR-1225-3p_st	-12363.531	-8.418278182	-19.86895169
hsa-miR-1225-5p_st	0.16738572	0.200627847	0.130470497
hsa-miR-1226_st	-4.100885095	-4.702071244	-122.7914531
hsa-miR-1226-star_st	-283.7951043	-1133.404019	0.3998387
hsa-miR-1227_st	-53.63383538	-1024.426117	0.017845754
hsa-miR-1228_st	0.06295357	0.048604375	0.140726268
hsa-miR-1228-star_st	0.346550029	-295.9879755	0.141865223
hsa-miR-1229_st	-23.68812228	0.268597574	-29.41109132
hsa-miR-122-star_st	-29.61761146	0.037723933	0.047031946
hsa-miR-1231_st	0.935603515	-2.51035716	0.216956598
hsa-miR-1233_st	0.957939818	0.152895858	0.573204953
hsa-miR-1234_st	-27.61602842	-145.9174347	-1.093324347
hsa-miR-1236_st	-7.481195354	0.013791449	-68.88076967
hsa-miR-1237_st	0.468388988	-37.37850259	0.372981972

hsa-miR-1238_st	-9.780075833	-21.40056441	0.110386408
hsa-miR-124_st	-6.628150481	-2.020551594	0.005358899
hsa-miR-1243_st	0.255465045	-87.92963945	0.200697728
hsa-miR-1244_st	0.191270212	0.593794307	0.189570112
hsa-miR-1245_st	0.13402262	0.22320475	0.060710496
hsa-miR-1246_st	0.235214815	0.13527022	0.674138703
hsa-miR-1247_st	0.326029009	-35.13741717	-0.359742954
hsa-miR-1248_st	-599.994975	-375.6220664	-13.66872729
hsa-miR-1249_st	-2.783676043	-1.931597675	-2.384329112
hsa-miR-124-star_st	0.007157921	0.020064864	0.07975795
hsa-miR-1250_st	0.063005769	1.169684429	0.098801777
hsa-miR-1251_st	-503.6164292	-50.60459978	0.047670609
hsa-miR-1252_st	0.0847625	-630.267438	0.241344739
hsa-miR-1253_st	0.082640726	0.268571661	0.00481407
hsa-miR-1254_st	0.038220055	8.182177063	5.91851584
hsa-miR-1255a_st	0.011157169	0.00016352	-3839.491114
hsa-miR-1255b_st	0.193489136	0.47734281	0.317059086
hsa-miR-1256_st	0.010166991	-9.326997107	0.014124015
hsa-miR-1257_st	0.109356868	0.047108532	0.005981553
hsa-miR-1258_st	-38.34879842	-13.44190828	-59.21121697
hsa-miR-1259_st	0.073684017	0.007064823	0.10741613
hsa-miR-125a-3p_st	-19.69798113	0.000696432	-14.61702545
hsa-miR-125a-5p_st	-2.156314749	0.000718508	-2.722505445
hsa-miR-125b_st	-13.8566626	-429.2747846	-28.69334312
hsa-miR-125b-1-star_st	-1.61986269	-0.635722807	-1.279419419
hsa-miR-125b-2-star_st	0.000174398	-3029.103044	0.185857125
hsa-miR-126_st	0.253516278	0.005902542	0.679232288
hsa-miR-1260_st	0.389004185	0.048712904	0.407745464
hsa-miR-1260b_st	-85496.39205	-1.991262202	0.051494502
hsa-miR-1261_st	0.051440281	-168.9937472	0.002389206
hsa-miR-1262_st	-22.04365533	-8.129875375	0.100127628
hsa-miR-1263_st	-12.4370598	-4.591279205	-7.396993913
hsa-miR-1264_st	-990.005377	0.035589766	0.000974876
hsa-miR-1265_st	-48.21861138	0.865189254	0.078995567
hsa-miR-1266_st	0.151895709	0.774192334	0.194585531
hsa-miR-1267_st	-297.2805369	-7.610596945	-28.54178393
hsa-miR-1268_st	0.172873424	0.439822966	0.071906031
hsa-miR-1269_st	-13389.71941	0.004970462	0.038797378
hsa-miR-126-star_st	0.001498309	-4.005173012	-8.194280519
hsa-miR-1270_st	0.125120314	0.46673218	0.447265351
hsa-miR-1271_st	0.005309162	-442.2072852	0.318023812
hsa-miR-1272_st	-9.562933717	-7.913051667	-5.379116947
hsa-miR-1273_st	0.003251965	0.010791885	0.129757728
hsa-miR-1273c_st	-21.36753649	0.052336628	-217.386816
hsa-miR-1273d_st	0.205884071	0.084351327	0.017416477
hsa-miR-127-3p_st	-4.49500249	-125.6473833	-7.400656732
hsa-miR-1274a_st	0.144788621	-71.02892356	0.435512224
hsa-miR-1274b_st	0.092776896	-32.76980482	0.26070368
hsa-miR-1275_st	-1.610949967	-49780.16085	-3.171026969
hsa-miR-127-5p_st	0.099308808	-5.857835668	0.561845441
hsa-miR-1276_st	0.015480834	-15.53407721	-25.95568011

hsa-miR-1277_st	-3.080204142	-32.282691	-42.99270937
hsa-miR-1278_st	-10.5714865	-7.463914164	-804.3320682
hsa-miR-1279_st	0.017540751	-169.2973247	0.003205428
hsa-miR-128_st	0.25538873	-2.154245403	0.16401366
hsa-miR-1280_st	-4.636041478	-0.354588628	-4.339274263
hsa-miR-1281_st	-106.43588	-14.40444232	-1.287045191
hsa-miR-1282_st	0.037439348	1.541345839	0.059495747
hsa-miR-1283_st	-9.317262941	-17.6865805	-6.899383573
hsa-miR-1284_st	-14.98257396	-3.6509405	-14.893949
hsa-miR-1285_st	0.006812604	-146.598421	0.176216508
hsa-miR-1286_st	-72.77554243	-8.730088821	0.000979408
hsa-miR-1287_st	0.033902384	-27.60268177	0.126088553
hsa-miR-1288_st	0.013876369	0.071325584	0.008596906
hsa-miR-1289_st	-10.42741091	-26.51860958	-11.20466069
hsa-miR-1290_st	0.601659295	0.39799885	0.427244596
hsa-miR-1291_st	-78.82795573	-657.816357	0.025080899
hsa-miR-1292_st	0.007644529	0.027521483	0.712472735
hsa-miR-1293_st	0.00795548	2.199655316	0.009603216
hsa-miR-129-3p_st	0.054519682	-52.66442213	0.010206253
hsa-miR-1294_st	0.026525985	0.683693325	0.118141032
hsa-miR-1295_st	-138.4767198	-238.5397166	-20.04135037
hsa-miR-129-5p_st	0.165675049	0.869362301	0.145518598
hsa-miR-1296_st	-26.31049485	-92.76577017	-7528.014551
hsa-miR-1297_st	0.229487465	0.090769036	0.275182603
hsa-miR-1298_st	0.049868026	0.003745073	-1558.889572
hsa-miR-1299_st	0.018033401	0.437299174	0.013844229
hsa-miR-129-star_st	-25.90708573	-107.2442466	-382.4476288
hsa-miR-1301_st	4.34742E-05	-3.327299033	0.556149029
hsa-miR-1302_st	0.062343848	0.003909251	0.197230583
hsa-miR-1303_st	-1535.881795	0.044387748	0.020278472
hsa-miR-1304_st	0.083113142	0.07240593	0.172274804
hsa-miR-1305_st	-18.53781894	-76.0926657	-52.9553195
hsa-miR-1306_st	-3.619908139	0.478832861	0.007658963
hsa-miR-1307_st	0.015068913	-4.07367436	-14.56319054
hsa-miR-1308_st	-58.89040085	-16.96369761	0.000775344
hsa-miR-130a_st	0.141841495	-5102.040816	0.036680102
hsa-miR-130a-star_st	-273.4041854	-522.8193688	-21.27460045
hsa-miR-130b_st	-9.527839438	-8.230311059	-14.86565187
hsa-miR-130b-star_st	-403.6325557	-5.282952834	-11.18480905
hsa-miR-132_st	-12.32016788	-7.021550654	-8.687068394
hsa-miR-1321_st	0.289722751	2.003109473	0.048304567
hsa-miR-1322_st	0.005560909	-5.717142036	-12472.85346
hsa-miR-1323_st	-3.602279425	-3.683553979	-1.753612153
hsa-miR-1324_st	-37.5058869	-6.383459408	-47.45763381
hsa-miR-132-star_st	-2.663107571	-2.242832918	-9.450582442
hsa-miR-133a_st	0.023706145	0.001177759	0.20543239
hsa-miR-133b_st	-6408.199871	-90.06108797	-29.86936449
hsa-miR-134_st	-2.846105623	-15.94280977	-31.99590765
hsa-miR-135a_st	-132.9447899	-4E+12	0.006652034
hsa-miR-135a-star_st	0.302748101	0.031529862	0.036929501
hsa-miR-135b_st	0.041583774	-204.9385492	-104.3126618

hsa-miR-135b-star_st	-53.77985725	-10.0019461	-17.65872034
hsa-miR-136_st	0.003942647	1.89791E-05	0.004737018
hsa-miR-136-star_st	0.080821373	0.023140951	0.121991279
hsa-miR-137_st	-12.2999115	-18.83310641	0.013651936
hsa-miR-138_st	0.064655267	-1.607530599	0.230882172
hsa-miR-138-1-star_st	-15.95695106	0.018542541	-1.152986065
hsa-miR-138-2-star_st	-52.92257926	0.132621246	-11.19120824
hsa-miR-139-3p_st	0.004813931	0.003873009	0.01333563
hsa-miR-139-5p_st	0.001116763	0.005973203	0.007641207
hsa-miR-140-3p_st	-137.401013	-27.3311939	0.002207073
hsa-miR-140-5p_st	0.177568689	-14.48812139	0.142672776
hsa-miR-141_st	0.007579618	-231.1487196	-11.37313777
hsa-miR-141-star_st	-83.65377686	-1.955306407	-19.12538143
hsa-miR-142-3p_st	0.000340347	-2.451531324	-6.380476749
hsa-miR-142-5p_st	-29.87867161	-3.094547281	-69.0055635
hsa-miR-143_st	0.057536897	0.07153191	0.204283661
hsa-miR-143-star_st	0.100138387	-5.689981219	0.012549489
hsa-miR-144_st	0.08099033	0.07531896	-55.94064129
hsa-miR-144-star_st	0.03609943	-8.376181443	0.043079493
hsa-miR-145_st	-25.99658923	0.144169432	-61.70145876
hsa-miR-145-star_st	-472.9290064	0.02908952	0.00024115
hsa-miR-1468_st	0.110775476	0.02402717	0.023109928
hsa-miR-1469_st	0.000416976	0.070984945	0.022034434
hsa-miR-146a_st	3.449228984	0.052279451	3.167616848
hsa-miR-146a-star_st	0.028390397	0.004038793	-8.808277536
hsa-miR-146b-3p_st	-160.8287025	-5.313955353	-44.77307052
hsa-miR-146b-5p_st	0.24114729	-5.089522482	0.473886988
hsa-miR-147_st	0.01972536	0.137925704	0.200263858
hsa-miR-1470_st	-0.506129777	0.02890272	-0.42110023
hsa-miR-1471_st	-1.390030907	0.977245931	-1.576822325
hsa-miR-147b_st	-194.4591766	-7.945494183	0.042701536
hsa-miR-148a_st	-3.569158657	-9.708322268	-32.74636859
hsa-miR-148a-star_st	-582.4729949	5.118788601	-174.5789375
hsa-miR-148b_st	-161.2374001	-2.822306073	-204.3559614
hsa-miR-148b-star_st	0.024111102	-1042.431381	-180.7903474
hsa-miR-149_st	-3.973856852	-13.37821632	-32.01926745
hsa-miR-149-star_st	0.151021618	-3042.316493	-36.68202016
hsa-miR-150_st	-32.93475419	-13.99007781	-27.85264038
hsa-miR-150-star_st	0.043654461	-5.563199368	0.012202295
hsa-miR-151-3p_st	-20.6733713	-23.3723051	0.044130245
hsa-miR-151-5p_st	-39.45805713	1.8297E-05	0.006530094
hsa-miR-152_st	0.025161098	0.063448572	0.290381955
hsa-miR-153_st	0.089554154	-66.05042073	-120437.7829
hsa-miR-1537_st	0.071666235	0.210397892	0.00196577
hsa-miR-1538_st	-466.4450609	0.097432316	-28.48256034
hsa-miR-1539_st	0.023413437	-57.36211419	0.016245032
hsa-miR-154_st	0.41261288	0.00089916	0.740665924
hsa-miR-154-star_st	0.361515391	0.038813728	0.879313172
hsa-miR-155_st	0.120336141	0.042578259	0.474534988
hsa-miR-155-star_st	0.027463284	0.001637902	0.041899634
hsa-miR-15a_st	0.536004819	-2.249312782	0.42635415

hsa-miR-15a-star_st	-15.28439163	0.006104688	-329.9063611
hsa-miR-15b_st	-4.881719481	-21.29443613	-1.001525744
hsa-miR-15b-star_st	0.016088766	0.020513257	-108.3297078
hsa-miR-16_st	0.234164049	0.000834343	0.003391315
hsa-miR-16-1-star_st	-1460.85906	-4.0488428	-8.775794896
hsa-miR-16-2-star_st	0.021035441	0.051062441	-38.76730865
hsa-miR-17_st	1.098045159	0.001318779	2.43290273
hsa-miR-17-star_st	0.337030175	-175.8918791	0.961510661
hsa-miR-181a_st	-1.249142884	0.013899231	-0.575858119
hsa-miR-181a-2-star_st	0.053223182	-2.25370131	0.489675254
hsa-miR-181a-star_st	0.373656514	0.014009918	0.516684941
hsa-miR-181b_st	-47.45240331	0.015947649	0.004249084
hsa-miR-181c_st	-18.61492107	-3.013045967	-4.2732033
hsa-miR-181c-star_st	-391.6064501	-817.6108429	0.001642276
hsa-miR-181d_st	0.070845404	0.722431152	0.029092249
hsa-miR-182_st	-404.1682972	0.08282625	5.296501797
hsa-miR-1825_st	-3.429139369	-0.933805706	-0.476028631
hsa-miR-1826_st	0.13140625	0.321993828	0.036295965
hsa-miR-1827_st	-21.26567363	-5595.026572	0.025803121
hsa-miR-182-star_st	-64.79426115	-35.96704664	0.042994852
hsa-miR-183_st	0.004523571	-14671.03089	0.310975753
hsa-miR-183-star_st	-1071.77969	-22.06063856	-17.76861018
hsa-miR-184_st	0.000251207	-97066174.86	0.026400075
hsa-miR-185_st	-10.17782873	-96.53474216	0.043624588
hsa-miR-185-star_st	0.247682396	1.495863533	0.573020234
hsa-miR-186_st	0.032885284	0.018501712	0.065144905
hsa-miR-186-star_st	0.002656887	0.231824916	0.007709279
hsa-miR-187_st	-46.74871173	-56.34701594	-625.4377298
hsa-miR-187-star_st	-8.315448031	0.118864974	-6.712876436
hsa-miR-188-3p_st	0.014493391	0.003071266	-7.0090642
hsa-miR-188-5p_st	-43.00680775	0.024965422	-1122.531738
hsa-miR-18a_st	1.559048159	-15.22677224	3.930788012
hsa-miR-18a-star_st	0.044068086	-68.65778872	-60.0045966
hsa-miR-18b_st	0.774177376	-10.51864948	1.534888077
hsa-miR-18b-star_st	-64.25934555	-250.0122796	-24.79082188
hsa-miR-190_st	0.16553628	0.270972303	0.294262309
hsa-miR-1908_st	0.52983841	-1601.921729	0.31152026
hsa-miR-1909_st	0.08221696	-47.70905933	0.041442373
hsa-miR-1909-star_st	0.38568385	-14.61747253	0.743317279
hsa-miR-190b_st	-1345.749505	0.048627968	0.004876089
hsa-miR-191_st	0.000952648	-265.5574703	0.07236907
hsa-miR-1910_st	-145.7378126	-2.47633005	-7.531353959
hsa-miR-1911_st	-18.86272767	0.140857221	-3.765081414
hsa-miR-1911-star_st	-53.24591391	-1605.904291	-5.55749586
hsa-miR-1912_st	0.010977905	0.022959826	0.001102207
hsa-miR-1913_st	0.000933058	0.007631158	-10.55613897
hsa-miR-1914_st	-2.801394909	-362.7496031	-0.252146942
hsa-miR-1914-star_st	-5.116971532	0.241010301	-4.805788435
hsa-miR-1915_st	0.239013432	0.033244229	0.137199864
hsa-miR-1915-star_st	-1.557076659	-8.601774247	-0.456147344
hsa-miR-191-star_st	0.719035314	-18.10620007	0.104978916

hsa-miR-192_st	0.479667056	0.000763058	1.319657579
hsa-miR-192-star_st	-5283.874884	-12.25448473	0.000493395
hsa-miR-193a-3p_st	-2.203007523	-9.757451423	-11.33121708
hsa-miR-193a-5p_st	-68.52088927	-2.292834556	-7.502110752
hsa-miR-193b_st	-3.516321637	-46.24366243	-5.28127754
hsa-miR-193b-star_st	0.002138045	0.001315912	0.122287642
hsa-miR-194_st	0.497177292	-85.9876766	2.065201801
hsa-miR-194-star_st	-27.09723162	-2486.554479	-18.27502009
hsa-miR-195_st	0.56814057	-49448.62557	0.466407727
hsa-miR-195-star_st	-69.56845287	-645.097037	0.102769292
hsa-miR-196a_st	0.011221377	0.383122004	0.20696176
hsa-miR-196a-star_st	-155.5880482	0.007375632	-11.84167844
hsa-miR-196b_st	0.004261609	0.4584204	0.008728324
hsa-miR-196b-star_st	0.005394756	4.601235212	0.106172683
hsa-miR-197_st	-86.19616587	1.4646385	0.122210022
hsa-miR-1972_st	0.970972759	0.316257767	1.965020811
hsa-miR-1973_st	0.466460997	0.043789957	0.501231433
hsa-miR-1975_st	-305.1047844	0.090778677	-6.591444747
hsa-miR-1976_st	-1.197520508	-3.132647723	-35.04906368
hsa-miR-1979_st	-4.786095475	-2.087515323	-2.594572453
hsa-miR-198_st	0.014848885	1.161263787	-4563.217653
hsa-miR-199a-3p_st	0.322263421	-115.9529612	0.023565627
hsa-miR-199a-5p_st	0.02722368	-397.1750517	-11346.28892
hsa-miR-199b-3p_st	0.522791903	-441.2417674	0.053084621
hsa-miR-199b-5p_st	0.036303967	-11.12972706	0.059855824
hsa-miR-19a_st	0.078464693	0.025580164	0.265274957
hsa-miR-19a-star_st	-488.0296044	-12.1585726	-68.46137231
hsa-miR-19b_st	6.266873874	0.004837481	6.306009925
hsa-miR-19b-1-star_st	-6.145576989	-7.100295612	-12.97805398
hsa-miR-19b-2-star_st	-218.0163917	-2.361353292	-18.84005541
hsa-miR-200a_st	-26.8028728	-16.84588788	-121.7624969
hsa-miR-200a-star_st	-144.9667608	-8.208631363	-24.58950048
hsa-miR-200b_st	0.05897418	-34.0615755	0.026666237
hsa-miR-200b-star_st	-20.35974343	-356.2136159	-810.5959466
hsa-miR-200c_st	0.044381428	0.019389727	0.190726105
hsa-miR-200c-star_st	-1.593858793	-4.812073633	-31.04693227
hsa-miR-202_st	-142.6626991	0.628775253	0.012419654
hsa-miR-202-star_st	0.016562146	-9.43998709	-36.91955343
hsa-miR-203_st	-6.492789652	0.002360502	-16.22245307
hsa-miR-204_st	-196.8565734	-128.8494861	-230.9029157
hsa-miR-205_st	-3.372020313	-2.9730873	-2.389366292
hsa-miR-2052_st	0.008413017	0.030418674	0.0379636
hsa-miR-2053_st	-4599.186206	-63.47183568	0.002675734
hsa-miR-2054_st	-1.284209937	-3.280981924	-0.823464143
hsa-miR-205-star_st	2.61837E-05	0.003237382	0.048233164
hsa-miR-206_st	-2674.64856	1.346448094	-9.090825712
hsa-miR-208a_st	-1267.936592	-26.50837048	0.052329765
hsa-miR-208b_st	-143046.4546	-40.58200343	-135.4270201
hsa-miR-20a_st	1.880976591	0.00226281	3.684756663
hsa-miR-20a-star_st	0.004987749	24.6466177	-23.19725121
hsa-miR-20b_st	2.035392996	-4.494344989	3.572449659

hsa-miR-20b-star_st	0.000752377	0.007646453	-23.17525681
hsa-miR-21_st	0.862540342	-23.15919948	0.976551098
hsa-miR-210_st	0.252596206	0.019879449	0.555364027
hsa-miR-211_st	0.167380402	0.00572996	0.006614162
hsa-miR-2110_st	-36.83870384	-15.835015	0.438959252
hsa-miR-2113_st	0.109902858	0.111669589	0.03836016
hsa-miR-2114_st	-355.0600621	0.001251497	2.08986E-05
hsa-miR-2114-star_st	-10.83927693	-27.14666829	-10.78789376
hsa-miR-2115_st	0.020216005	-421.6056354	-168448.508
hsa-miR-2115-star_st	0.000824609	-57.80123961	0.036193921
hsa-miR-2116_st	-64.20220589	0.024790188	-52.55795364
hsa-miR-2116-star_st	-1236.043994	-8.723772585	-10.61174756
hsa-miR-2117_st	0.018503617	-14.31986033	0.012144811
hsa-miR-212_st	0.098548906	-19.56083324	0.07034934
hsa-miR-214_st	-0.743530775	-3.131478148	-0.878691507
hsa-miR-214-star_st	0.185358664	-13.9747062	0.811216356
hsa-miR-215_st	-116.7221579	0.016756267	0.000342361
hsa-miR-216a_st	-56.41602286	-28.35424413	-3.972455082
hsa-miR-216b_st	-14.46581284	-4.264797445	-12.15920857
hsa-miR-217_st	-12.29318485	-4.572018465	-2.212665516
hsa-miR-218_st	0.086913526	-3.125093085	0.067045263
hsa-miR-218-1-star_st	-6.589955798	-21.14050819	-1.407738829
hsa-miR-218-2-star_st	-3.963160435	-3.069302405	-1.121294713
hsa-miR-219-1-3p_st	0.10209335	0.053148461	0.285486642
hsa-miR-219-2-3p_st	0.001123423	-906.8063638	0.001547832
hsa-miR-219-5p_st	-10.47986644	-2.106965241	-83.88224891
hsa-miR-21-star_st	1.256747497	0.649722245	0.738483282
hsa-miR-22_st	0.160505599	0.316126749	0.16723724
hsa-miR-220a_st	0.138910508	0.005202665	0.035975278
hsa-miR-220b_st	-37.66996524	-10.07126209	-29.76029021
hsa-miR-220c_st	-6.242506745	4.56976E-07	-13.76550735
hsa-miR-221_st	-67.05975654	-2654.293412	-42.6321898
hsa-miR-221-star_st	0.181581237	-53546.48475	0.20032159
hsa-miR-222_st	-7.04146396	-103.6890272	-5.64820517
hsa-miR-222-star_st	0.710840374	-2.824440911	0.134181551
hsa-miR-223_st	-776.0176367	-8.431728038	-35.94419297
hsa-miR-223-star_st	-343.5842141	-423.0289516	0.031184205
hsa-miR-224_st	0.011084721	-38.59433853	0.023072547
hsa-miR-224-star_st	-427.6429335	-13.74571254	-4.392750778
hsa-miR-2276_st	0.0105986	0.156446354	0.063788827
hsa-miR-2277_st	0.5162092	-4.020357376	0.478014601
hsa-miR-2278_st	0.2211487	1.829687723	-484.8432603
hsa-miR-22-star_st	0.346638338	0.092084937	0.845582318
hsa-miR-2355_st	3.16744E-05	-5.280537266	-22.23952435
hsa-miR-23a_st	0.019989718	0.00028968	0.010064102
hsa-miR-23a-star_st	-1.333419259	0.014554251	-1.440459182
hsa-miR-23b_st	0.006130107	0.014537125	0.037156418
hsa-miR-23b-star_st	-93.3095945	0.028024267	0.365095101
hsa-miR-24_st	0.004397679	0.003524203	0.117697025
hsa-miR-24-1-star_st	0.021964722	-10.5336076	-3.200017109
hsa-miR-24-2-star_st	0.332219092	-92.73986487	0.719262585

hsa-miR-25_st	0.045462342	-1.524995594	0.05333721
hsa-miR-25-star_st	0.012062409	-1.901112241	0.173348572
hsa-miR-26a_st	0.089200782	-1968.304007	0.026817338
hsa-miR-26a-1-star_st	0.0189002	0.21239747	0.000751198
hsa-miR-26a-2-star_st	0.119302196	0.127047335	0.033167877
hsa-miR-26b_st	0.200326066	-44.91029716	0.111991626
hsa-miR-26b-star_st	0.010194941	0.007269438	-14926.65972
hsa-miR-27a_st	0.00651919	0.324983795	0.086300225
hsa-miR-27a-star_st	0.180428703	-3.177536403	0.543484482
hsa-miR-27b_st	-369.3677389	-49.81198629	0.300354418
hsa-miR-27b-star_st	-69.67941404	-3.65110793	0.742139176
hsa-miR-28-3p_st	-74.60284952	-1.581344542	0.019160789
hsa-miR-28-5p_st	-14.34476535	-8.783625345	-102.0438043
hsa-miR-2861_st	0.674320169	0.013948791	0.446765244
hsa-miR-2909_st	-10.01927128	-5096.214907	0.008253268
hsa-miR-296-3p_st	0.004640743	1.096835195	0.313939452
hsa-miR-296-5p_st	0.026583183	-26.40386254	0.07569294
hsa-miR-297_st	0.067493442	1.221794201	-4.834785729
hsa-miR-298_st	-520.7811467	0.010391866	0.011894847
hsa-miR-299-3p_st	0.543589909	-10.16077097	0.445355355
hsa-miR-299-5p_st	-2571.367533	-1.285299198	-25.60592138
hsa-miR-29a_st	0.001666272	-273.9746876	-4.011850266
hsa-miR-29a-star_st	-117.6379855	0.042218126	-10.77691799
hsa-miR-29b_st	0.711783287	-7.569611872	0.928530742
hsa-miR-29b-1-star_st	-59.96046388	-0.447058886	-2.238881378
hsa-miR-29b-2-star_st	-457.5187541	-18.36637613	6.1009E-08
hsa-miR-29c_st	0.03503654	-1.949190827	0.015195863
hsa-miR-29c-star_st	-326.2930602	-680.5056306	-100.4968438
hsa-miR-300_st	0.01036996	0.027668996	0.013565261
hsa-miR-301a_st	0.074405655	-197.6794606	-5502.445917
hsa-miR-301b_st	0.066813203	-9.882291128	-91.49067878
hsa-miR-302a_st	-48.70984303	0.004829625	1.200224325
hsa-miR-302a-star_st	0.002506204	0.015298474	0.170674331
hsa-miR-302b_st	0.021529106	0.08513907	0.427244596
hsa-miR-302b-star_st	-4.906687297	-2967.540018	-339.0005575
hsa-miR-302c_st	-16.61127983	-15.81958819	-12.23203678
hsa-miR-302c-star_st	0.003428278	0.516664814	1.746678103
hsa-miR-302d_st	-5.540868244	0.384362801	2.341562537
hsa-miR-302d-star_st	-8.471679754	-415.1449321	0.012201964
hsa-miR-302e_st	0.007596778	0.004612923	-13.68805212
hsa-miR-302f_st	-15.91623435	0.026364991	0.00181493
hsa-miR-3065-3p_st	-186.7585412	-156.5179222	-6.443521804
hsa-miR-3065-5p_st	0.19855936	-653.0675532	0.01584804
hsa-miR-3074_st	-50.11535597	-11.16967494	-133.3519348
hsa-miR-30a_st	0.762006785	0.016088132	0.634354107
hsa-miR-30a-star_st	2.54318E-05	-0.837891372	-5.875526875
hsa-miR-30b_st	0.440182489	-9.2106263	0.730284757
hsa-miR-30b-star_st	-18407.97852	0.090635016	0.133429478
hsa-miR-30c_st	0.554613836	0.078236286	0.600414993
hsa-miR-30c-1-star_st	-7.323531867	-29.49036431	-58.33666081
hsa-miR-30c-2-star_st	0.005466236	-793.7392828	0.349362563

hsa-miR-30d_st	-671.0372611	-516.575886	0.041181194
hsa-miR-30d-star_st	0.02912347	-35200.23655	-136.2470972
hsa-miR-30e_st	0.300772723	-27.37597199	0.300893937
hsa-miR-30e-star_st	0.003301652	-4.129543976	-22.20380372
hsa-miR-31_st	0.000195021	0.001577678	-13.33443165
hsa-miR-3115_st	0.027467593	-439.9654737	0.018796958
hsa-miR-3116_st	-5.45006201	0.051663244	-333.4155769
hsa-miR-3117_st	0.147615403	-7028.533823	0.014873754
hsa-miR-3118_st	0.126546679	0.359055021	0.215541526
hsa-miR-3119_st	0.012945661	0.013771023	0.270226868
hsa-miR-3120_st	0.000729864	0.009919961	0.040433569
hsa-miR-3121_st	-417.6003122	0.07921551	0.01802615
hsa-miR-3122_st	0.019951704	0.019543621	0.001699047
hsa-miR-3123_st	0.016882195	0.120752775	-144.7244501
hsa-miR-3124_st	0.923402801	-4.32890694	0.462731219
hsa-miR-3125_st	-1647.49594	0.424838026	0.166205428
hsa-miR-3126-3p_st	0.283319869	0.030178638	0.117181955
hsa-miR-3126-5p_st	-22.60560528	-11.61677832	-22.00087019
hsa-miR-3127_st	-10.32566503	0.686950052	-17.28389568
hsa-miR-3128_st	0.248307375	-10.83670798	0.001751757
hsa-miR-3129_st	-54.37796779	0.223160343	0.004432364
hsa-miR-3130-3p_st	-29.36377633	-53.36188625	-47.71301399
hsa-miR-3130-5p_st	0.00063011	0.007901432	0.011352902
hsa-miR-3131_st	-16.05113022	0.011179573	0.19009382
hsa-miR-3132_st	-10.77228488	0.179615764	-57.82101976
hsa-miR-3133_st	-976.196392	-2.66242539	-5.162461248
hsa-miR-3134_st	-17.98996346	-21.17603146	-83.32346227
hsa-miR-3135_st	-594.5069335	-14.04455235	-13.24572084
hsa-miR-3136_st	-19.66087827	-49.04231439	-87.41411679
hsa-miR-3137_st	-9.801091185	1.529640847	-1730.986489
hsa-miR-3138_st	0.105974338	6.590194944	0.065711221
hsa-miR-3139_st	0.09508589	0.104515778	0.043801048
hsa-miR-3140_st	-12.80316016	-4.940994267	0.00027879
hsa-miR-3141_st	0.087921738	0.011281308	-135.3923546
hsa-miR-3142_st	-71.95374992	-180.0463724	-52.10932728
hsa-miR-3143_st	-17.60334486	-2.194371242	-2.015860963
hsa-miR-3144-3p_st	0.060906044	-13.98280712	0.364187507
hsa-miR-3144-5p_st	-100.9466688	0.003703331	-1553.120004
hsa-miR-3145_st	-6.806743061	0.010596541	-8.460274722
hsa-miR-3146_st	0.000278206	0.041839066	3.00852E-05
hsa-miR-3147_st	0.002543487	16.99789212	-35.64204374
hsa-miR-3148_st	0.026511329	1.449838749	0.011245754
hsa-miR-3149_st	-9.469465955	1.92384806	-129.8868374
hsa-miR-3150_st	0.001842298	-90.3626923	0.046688406
hsa-miR-3151_st	0.020106247	1.661742715	0.135075361
hsa-miR-3152_st	0.019288349	0.006798415	0.003861939
hsa-miR-3153_st	-3.959872462	-151.1052476	-3.941341148
hsa-miR-3154_st	-56.09325024	10.18479142	-25.13038278
hsa-miR-3155_st	-154.6298961	-22.79868365	-1103.410629
hsa-miR-3156_st	-4.433318214	0.448082303	-4.167116602
hsa-miR-3157_st	-44.06584355	-22.28837338	-4.303699632

hsa-miR-3158_st	0.016127984	0.002239892	-10136.37928
hsa-miR-3159_st	0.123244528	0.106820137	0.0269467
hsa-miR-3160_st	0.021001082	0.003073926	0.046481635
hsa-miR-3161_st	-32.19720247	2.18089E-07	-4.160497894
hsa-miR-3162_st	-10.50981937	2.808077736	-18.02932432
hsa-miR-3163_st	-5754.021706	0.000582185	0.016445498
hsa-miR-3164_st	-30.40421175	-2.059598038	-2.528697572
hsa-miR-3165_st	-8.568494541	-5.090533044	-21.22161249
hsa-miR-3166_st	-39.44913575	-2.665038214	-15.0940175
hsa-miR-3167_st	-92.54903665	-4.866310546	-6.361212516
hsa-miR-3168_st	0.056831699	0.020625843	0.322357663
hsa-miR-3169_st	-126.9131724	-14.03476765	0.000163009
hsa-miR-3170_st	0.021042839	0.190831807	-337.0800808
hsa-miR-3171_st	0.008356885	-1632.420524	0.012831792
hsa-miR-3172_st	-13.61421205	-29.74600844	0.094132376
hsa-miR-3173_st	-42.00372925	0.319728048	0.084286573
hsa-miR-3174_st	-84.61298938	-88.09808383	0.038418548
hsa-miR-3175_st	0.575240335	0.603592256	1.537438804
hsa-miR-3176_st	-115.7883218	0.01629682	-52.66747976
hsa-miR-3177_st	0.015531017	0.065558786	0.132785902
hsa-miR-3178_st	-15.3612259	-1.893475974	-3.771335564
hsa-miR-3179_st	0.005276788	0.015475609	0.069222662
hsa-miR-3180-3p_st	0.663350793	-1.624345779	0.413249694
hsa-miR-3180-5p_st	-0.40050679	0.059092019	-0.106384773
hsa-miR-3181_st	0.066235456	-2.531381783	0.052379875
hsa-miR-3182_st	-3056.461301	0.722691263	0.084845495
hsa-miR-3183_st	-56.90736238	-85.20211947	-77.35674176
hsa-miR-3184_st	0.000393685	-17.29216207	-71.27740321
hsa-miR-3185_st	0.827817924	-43.92460509	0.002379488
hsa-miR-3186-3p_st	1.99898E-05	0.681956375	0.124773552
hsa-miR-3186-5p_st	0.037216969	0.006756676	-8.141756882
hsa-miR-3187_st	1.189263315	-5.464557333	0.763128913
hsa-miR-3188_st	0.619162823	-2.946385734	-29.77019611
hsa-miR-3189_st	0.091942065	1.789339626	0.041400244
hsa-miR-3190-3p_st	-130.797538	0.000615164	0.001877576
hsa-miR-3190-5p_st	-741.9456295	0.046537491	-6.770166324
hsa-miR-3191_st	-24.00238584	-6.174702887	-2808.08449
hsa-miR-3192_st	0.108509301	2.059847837	0.263980164
hsa-miR-3193_st	-5.752312344	-22.85146291	-32.33962744
hsa-miR-3194_st	0.006901871	-6.324757782	-1.138245169
hsa-miR-3195_st	-3.366503772	-2.10035646	-2.929546041
hsa-miR-3196_st	0.095298777	-37.8535146	0.155208421
hsa-miR-3197_st	0.160431891	-997.9651242	-6980.463588
hsa-miR-3198_st	-17.89556741	0.887347986	-32.33962744
hsa-miR-3199_st	0.13552706	0.122225054	0.184945153
hsa-miR-31-star_st	2.319417822	-2.241933007	0.740930158
hsa-miR-32_st	0.187210252	-22.04955582	0.151049988
hsa-miR-3200_st	0.210475877	0.040841176	0.31448262
hsa-miR-3201_st	-4.772168618	-0.38144483	-0.505071128
hsa-miR-3202_st	0.062408783	0.068956397	0.154417562
hsa-miR-320a_st	-1.945595486	-9.913149696	-2.210149782

hsa-miR-320b_st	-1.491280955	-7.204754837	-2.012171014
hsa-miR-320c_st	-1.666009182	-11.19266848	-1.825647154
hsa-miR-320d_st	-1.677630092	-7.332478407	-2.981018276
hsa-miR-320e_st	0.28498995	-17.4748761	0.304347512
hsa-miR-323-3p_st	0.011410085	-5.025433405	0.059045112
hsa-miR-323-5p_st	-1754.117455	-222.1562436	-51.86157217
hsa-miR-323b-3p_st	0.001073676	-28.83573531	0.113667425
hsa-miR-323b-5p_st	-140.8534208	0.029972958	0.002337142
hsa-miR-324-3p_st	-17.29007694	-13.32761731	0.034802395
hsa-miR-324-5p_st	-5.394081565	-2.492766467	-14.84881528
hsa-miR-325_st	0.102943118	0.475826109	0.166582749
hsa-miR-326_st	0.1501702	-100.8250707	0.146194964
hsa-miR-328_st	-8.553990682	-5.024160616	-47.65144298
hsa-miR-329_st	0.629255875	-4.419117234	0.755566878
hsa-miR-32-star_st	-7.193802259	0.390555003	-29.9169254
hsa-miR-330-3p_st	0.594536608	-9.686368283	1.44743397
hsa-miR-330-5p_st	-15.36929667	0.191255342	-1460.468288
hsa-miR-331-3p_st	-34.570752	-7.83589032	0.005366954
hsa-miR-331-5p_st	-105.6322759	-1.765442931	0.00151718
hsa-miR-335_st	0.001302488	-44.24305736	0.033662892
hsa-miR-335-star_st	-1947.2544	0.005880129	0.00021354
hsa-miR-337-3p_st	-6.976776978	-13.93742841	-2.183857686
hsa-miR-337-5p_st	0.0484066	-6.606871475	0.339652343
hsa-miR-338-3p_st	-4.525394574	-7.001629067	-4.804303302
hsa-miR-338-5p_st	-34.61917996	-131.6075652	0.001310874
hsa-miR-339-3p_st	1.114310361	0.089788325	1.710106752
hsa-miR-339-5p_st	0.003071044	-2.365659086	0.288266442
hsa-miR-33a_st	-60.71976398	0.000781482	-32.40280056
hsa-miR-33a-star_st	0.037054325	0.009838061	-324.6021821
hsa-miR-33b_st	0.021570503	0.102329932	-41.39731931
hsa-miR-33b-star_st	-32.74880479	-3.601534307	0.009579711
hsa-miR-340_st	0.064086441	-8.458946045	0.000131309
hsa-miR-340-star_st	-332.3830115	-30767.90444	-11.28065673
hsa-miR-342-3p_st	-5.975744199	-8.62964299	-5.140083703
hsa-miR-342-5p_st	0.03198303	-5.131215251	0.006592222
hsa-miR-345_st	-96.65626145	-0.890638256	-12.01036864
hsa-miR-346_st	-74.10596126	-1.16485191	-5325.601724
hsa-miR-34a_st	-53127.67328	-25.44998489	-15.9147105
hsa-miR-34a-star_st	2.439331691	-43.37409936	1.027204547
hsa-miR-34b_st	0.050083083	0.427334803	0.10790798
hsa-miR-34b-star_st	-10.77398216	-4.318343399	-129.2746881
hsa-miR-34c-3p_st	0.004678355	-29.69723593	0.132795013
hsa-miR-34c-5p_st	0.04181432	-33.36918903	0.233344548
hsa-miR-361-3p_st	0.00106599	-21.17427753	-9838.97124
hsa-miR-361-5p_st	-67.31639589	-22.8439274	0.004807758
hsa-miR-362-3p_st	0.068564637	-37124.8993	-24.70697631
hsa-miR-362-5p_st	0.166685618	-19.7948535	0.683293185
hsa-miR-363_st	0.054216794	-18.46516155	0.12361377
hsa-miR-363-star_st	-3.935799308	-6.593916164	-54.53106784
hsa-miR-365_st	-20.11611644	-18.40745332	0.054607043
hsa-miR-365-star_st	-77.3744345	-2.456132418	-14194.29446

hsa-miR-367_st	0.047551908	-30.29836717	-85296.75081
hsa-miR-367-star_st	-16.50053378	0.012984147	-6457.731666
hsa-miR-369-3p_st	-10.12156523	0.051091595	0.031402144
hsa-miR-369-5p_st	0.557909653	0.027267091	0.651645297
hsa-miR-370_st	-1.020590621	-3.865330765	-1.674505454
hsa-miR-371-3p_st	-55.96700978	1.70404E-05	0.056162838
hsa-miR-371-5p_st	-121.0215409	0.183143342	0.030924278
hsa-miR-372_st	-633.12525	0.008667703	0.020966461
hsa-miR-373_st	0.055286117	1.545700401	-747.5550929
hsa-miR-373-star_st	0.146625429	0.012904392	0.1395467
hsa-miR-374a_st	0.012748329	-7.542258101	0.054341438
hsa-miR-374a-star_st	0.09785166	-8348.494181	0.237625538
hsa-miR-374b_st	0.034801089	-69.05660895	-100.8432964
hsa-miR-374b-star_st	0.00081736	0.067348814	0.151038718
hsa-miR-375_st	-43.98521928	0.024108773	0.017294354
hsa-miR-376a_st	2.604076101	-9.782492522	2.117455702
hsa-miR-376a-star_st	0.006261478	-8.587462651	-115.4154428
hsa-miR-376b_st	0.046371531	-80.09008956	0.064682223
hsa-miR-376c_st	0.879059069	0.003327155	0.944315554
hsa-miR-377_st	0.001780418	0.057163072	0.006341415
hsa-miR-377-star_st	0.059306861	-4.440860806	0.035556571
hsa-miR-378_st	3.49691309	0.009628123	5.447236247
hsa-miR-378b_st	-27.10075833	-4.113453791	-17.63388756
hsa-miR-378c_st	0.860732123	0.018512051	2.765203152
hsa-miR-378-star_st	-67622.98986	-97.55766782	-77267.77292
hsa-miR-379_st	0.143863559	0.013607106	0.450745062
hsa-miR-379-star_st	0.397226407	0.045412036	0.185687306
hsa-miR-380_st	0.017120676	0.009158777	-263.9334867
hsa-miR-380-star_st	-18.73119559	-1.804388698	-410.3651911
hsa-miR-381_st	0.311838481	-53.91222282	0.806428246
hsa-miR-382_st	-742.936893	0.002011747	0.032352138
hsa-miR-383_st	0.022441089	0.034716447	0.023973722
hsa-miR-384_st	0.00609149	-400.977789	0.014855222
hsa-miR-409-3p_st	0.00033206	-26.04051835	0.10816633
hsa-miR-409-5p_st	-691.5922746	-5.953500219	0.186971057
hsa-miR-410_st	0.001390209	-3.468166624	0.085549523
hsa-miR-411_st	1.239602777	-26272.41907	1.647723948
hsa-miR-411-star_st	0.189791794	0.060827343	0.691605962
hsa-miR-412_st	-4.335164389	-1.698460509	-1.565402475
hsa-miR-421_st	0.095518084	-3.542319028	0.860630073
hsa-miR-422a_st	0.451426766	0.595259312	1.495408591
hsa-miR-423-3p_st	-1.625580337	-5.497122996	-2.943141512
hsa-miR-423-5p_st	-2.725679668	-9.146253712	-2.018981452
hsa-miR-424_st	0.006266622	-60.47776589	0.070482816
hsa-miR-424-star_st	-0.677166065	-0.823175779	-0.771348064
hsa-miR-425_st	0.172679725	0.081631061	0.334542511
hsa-miR-4251_st	-6812.107153	0.025097847	0.00073563
hsa-miR-4252_st	0.044821336	0.083927828	0.112804962
hsa-miR-4253_st	-233.5785104	0.000269009	0.135654466
hsa-miR-4254_st	-10.08367458	0.093406946	-16.7005995
hsa-miR-4255_st	-1253.700287	0.155996956	5.4339E-05

hsa-miR-4256_st	0.060011771	0.011155796	0.022099201
hsa-miR-4257_st	0.063239927	0.989950376	0.026208048
hsa-miR-4258_st	-5.16454976	-3.608214435	-10.40277332
hsa-miR-4259_st	0.039620106	3.431098644	0.046394791
hsa-miR-425-star_st	0.345439483	-7.633850222	0.143338717
hsa-miR-4260_st	0.452482238	-28.04694825	0.278422965
hsa-miR-4261_st	-489.7591887	0.000322975	-125.405486
hsa-miR-4262_st	0.069411699	0.056970768	0.135843108
hsa-miR-4263_st	0.00049883	-32.7825647	-149.252009
hsa-miR-4264_st	0.007272167	0.18393977	0.037673645
hsa-miR-4265_st	0.009837267	0.296789062	0.085116312
hsa-miR-4266_st	-17.82725161	-104.8968026	-52.59531436
hsa-miR-4267_st	0.026451607	0.239569134	0.163167928
hsa-miR-4268_st	-77.36626786	-11.19678863	0.059342178
hsa-miR-4269_st	-1805.69922	0.200968407	0.336668013
hsa-miR-4270_st	-74.0747121	0.182198788	-1.648565822
hsa-miR-4271_st	0.008994531	0.898432892	0.009069991
hsa-miR-4272_st	-202.7471057	-6.947623157	0.016400516
hsa-miR-4273_st	0.101003832	0.366801021	-83.04729569
hsa-miR-4274_st	0.057032843	-0.726036272	-8.124940143
hsa-miR-4275_st	0.049247377	-870.5481372	0.157945836
hsa-miR-4276_st	-14.84280914	0.525907063	0.000152017
hsa-miR-4277_st	0.089537994	0.603742986	0.225701957
hsa-miR-4278_st	-409.8668677	-134.6190105	0.000584092
hsa-miR-4279_st	-5203.755485	0.01684804	-14.5379365
hsa-miR-4280_st	-171.6499235	-32.38344221	0.001923173
hsa-miR-4281_st	0.054513144	-74.77260714	-743.9910204
hsa-miR-4282_st	-4.57571604	-6.551872288	-3.307847696
hsa-miR-4283_st	0.000865889	0.010215145	-10.92319546
hsa-miR-4284_st	1.559005706	-63.51888961	2.235594631
hsa-miR-4285_st	0.019287793	-2.533404805	0.05522923
hsa-miR-4286_st	0.026271709	-369.7087199	0.028344079
hsa-miR-4287_st	-9.258641384	-231.5851081	-411.0727064
hsa-miR-4288_st	0.022953614	0.019465551	0.089966403
hsa-miR-4289_st	0.005288417	0.000499522	0.001858817
hsa-miR-429_st	-52.86948914	-6.403456773	-918.3014727
hsa-miR-4290_st	-26.41417689	0.826933791	0.070551859
hsa-miR-4291_st	0.030639527	0.667227586	0.020482189
hsa-miR-4292_st	0.052997105	-4.706142126	0.078316862
hsa-miR-4293_st	0.08558521	0.001026465	7.77852E-06
hsa-miR-4294_st	0.016595108	0.048699662	0.161871038
hsa-miR-4295_st	0.094075011	0.078752916	0.157682056
hsa-miR-4296_st	-9.324490953	0.463165997	-38.31652148
hsa-miR-4297_st	0.336979089	0.582663529	0.519540386
hsa-miR-4298_st	-0.360887959	-49.41917031	-0.13460833
hsa-miR-4299_st	0.020622683	0.001800475	-146.3219156
hsa-miR-4300_st	-28942.81952	0.304661497	0.062652092
hsa-miR-4301_st	0.003455617	-4.182333839	-9.44648733
hsa-miR-4302_st	-1416.820945	-45.4959265	0.001285473
hsa-miR-4303_st	0.230244986	0.922811918	0.189556615
hsa-miR-4304_st	0.089759561	0.02156316	0.050705807

hsa-miR-4305_st	-4.504624193	-2.853851807	-68.02054186
hsa-miR-4306_st	0.00557098	0.101088705	-1085.739252
hsa-miR-4307_st	0.023796148	0.310096958	0.068412064
hsa-miR-4308_st	0.389949667	0.243411983	0.189269808
hsa-miR-4309_st	0.005880359	-347.2886339	-7.250533988
hsa-miR-431_st	-6.696418633	-233.3288216	0.000741391
hsa-miR-4310_st	-10.3631274	0.010544209	-7.014690127
hsa-miR-4311_st	-5.714750537	0.058057143	-8.135604025
hsa-miR-4312_st	0.050144197	-69.79763786	0.04460016
hsa-miR-4313_st	-1.755934338	-0.762797935	-1.422228978
hsa-miR-4314_st	0.002013407	0.265755715	0.111039567
hsa-miR-4315_st	0.03476323	0.1404578	0.013120328
hsa-miR-4316_st	-149.0807576	-8941.248822	4.3514E-06
hsa-miR-4317_st	0.005752692	-7.878064835	0.079711358
hsa-miR-4318_st	0.063541554	0.779212253	-2269.19426
hsa-miR-4319_st	-72.01787968	-18.61307399	-93.17723824
hsa-miR-431-star_st	-3.358629745	0.000229629	-12.20905319
hsa-miR-432_st	-7.584169536	0.01172445	0.012357546
hsa-miR-4320_st	-67.89393368	0.002680806	0.00025802
hsa-miR-4321_st	0.179053807	-12.02603424	0.125713503
hsa-miR-4322_st	-302.431168	0.172192217	0.021907552
hsa-miR-4323_st	2.21323E-05	-48.53591184	0.0121219
hsa-miR-4324_st	0.002512766	0.030883493	0.189024518
hsa-miR-4325_st	-154878.3345	0.035975468	0.014854247
hsa-miR-4326_st	-11.97444544	-127.7912801	-5.01726444
hsa-miR-4327_st	0.457920183	0.9691103	0.014194578
hsa-miR-4328_st	-318.8889398	0.001698223	-609875.0046
hsa-miR-4329_st	-45.06690745	-3.626680915	0.036710177
hsa-miR-432-star_st	-7.920157239	-14.329782	0.032717213
hsa-miR-433_st	-0.746432677	-0.678905213	-2.194741859
hsa-miR-4330_st	0.068817291	0.360292259	-3364.733472
hsa-miR-448_st	0.000783664	-76.24955638	0.008609149
hsa-miR-449a_st	0.108657255	0.348609356	0.295881059
hsa-miR-449b_st	0.090635016	-69.21699543	0.110913643
hsa-miR-449b-star_st	0.090088522	1.091946626	-66.65086987
hsa-miR-449c_st	-16.51737032	0.045167938	-8.989497201
hsa-miR-449c-star_st	0.030990258	0.099651021	-7.468075777
hsa-miR-450a_st	-19.6029464	-63.64716166	0.020483621
hsa-miR-450b-3p_st	-472.9804343	-28.23459293	-80.07145729
hsa-miR-450b-5p_st	-5.744924633	-1.302997851	-2.175583936
hsa-miR-451_st	-56.60250759	-13.6852166	-260.0575901
hsa-miR-452_st	-52.2574735	-8.738968916	-12.9735668
hsa-miR-452-star_st	0.000219114	-129.5632591	0.000828317
hsa-miR-454_st	0.007331812	-20.96259386	0.001454812
hsa-miR-454-star_st	-49.95043775	0.007432854	-16.52946021
hsa-miR-455-3p_st	-36.21704855	-3694.334376	0.002214173
hsa-miR-455-5p_st	0.092288668	-908.8578529	0.29949898
hsa-miR-466_st	-95.17865388	3.676064215	-9.29670632
hsa-miR-483-3p_st	-35.81074151	-4.124940751	-5.525626786
hsa-miR-483-5p_st	-3.86563476	1.544658723	-43.04038978
hsa-miR-484_st	0.231128255	0.005462171	0.203833739

hsa-miR-485-3p_st	-14.21975992	-67.13615395	0.079764445
hsa-miR-485-5p_st	-2.866447521	-0.94443804	-8.523823906
hsa-miR-486-3p_st	0.247390843	0.003324906	0.031855646
hsa-miR-486-5p_st	-38.70896102	0.000860513	0.53582766
hsa-miR-487a_st	0.00490077	-2.65466616	0.09329115
hsa-miR-487b_st	-24.30064842	0.008593197	0.012085374
hsa-miR-488_st	-645.638071	-15.65998114	-210.7785116
hsa-miR-488-star_st	0.198100657	0.035097212	-48.97232673
hsa-miR-489_st	-7.268862069	-12.30172347	-88.41231467
hsa-miR-490-3p_st	0.006694594	-4.378450926	0.082259116
hsa-miR-490-5p_st	1.84857E-05	-4.271578409	0.003825484
hsa-miR-491-3p_st	-28.38658206	-2296.571484	-12.34103793
hsa-miR-491-5p_st	-23.14972902	-3.580872915	-18.13937468
hsa-miR-492_st	-13.28761575	-26.59662094	-18.45968783
hsa-miR-493_st	-4.971392013	-1.941003728	-13.39290816
hsa-miR-493-star_st	-28.2584625	-3.802463116	-4.113462134
hsa-miR-494_st	0.137316937	0.056915883	0.30534963
hsa-miR-495_st	0.992259039	-495.4994341	0.825199644
hsa-miR-496_st	0.08362941	-13.2520865	0.142713195
hsa-miR-497_st	-51.02441715	-167621.9371	0.011835155
hsa-miR-497-star_st	0.002508207	0.000466279	-82.77551213
hsa-miR-498_st	-3936.20636	-5.389912212	0.0886634
hsa-miR-499-3p_st	-11.33988051	-5.091003977	0.007979402
hsa-miR-499-5p_st	-84.50413039	0.08038274	-48.27927246
hsa-miR-500_st	0.007051549	-2.01393898	0.083901467
hsa-miR-500b_st	-8504.739827	-34.39761324	0.000859692
hsa-miR-500-star_st	-23.69008235	-3.37404603	0.003404956
hsa-miR-501-3p_st	0.001125703	-3.890216775	0.000672054
hsa-miR-501-5p_st	0.068682519	-4.315778039	0.455193777
hsa-miR-502-3p_st	-11.89741569	-11.19427881	0.066033838
hsa-miR-502-5p_st	-341.0697321	-18.1297987	0.021290604
hsa-miR-503_st	-3.209977826	-1.90800389	-4.286022907
hsa-miR-504_st	-204.929748	-226.571939	-14.05218732
hsa-miR-505_st	-7.900490008	-14.80177914	0.037629598
hsa-miR-505-star_st	-31734.56474	-1.522105188	0.890878307
hsa-miR-506_st	0.183733536	0.008525845	0.001676247
hsa-miR-507_st	-364.2187425	0.00122542	-6076.900108
hsa-miR-508-3p_st	0.030757268	0.0159301	-27.93041571
hsa-miR-508-5p_st	-1272455.256	6.50442E-05	-141.7182946
hsa-miR-509-3-5p_st	-2099.382126	-7.298091339	-54.53751137
hsa-miR-509-3p_st	0.106454723	0.474032939	0.153690657
hsa-miR-509-5p_st	-17.2559776	-4.325252521	-10.1275573
hsa-miR-510_st	0.137462978	0.012371334	-19.86602936
hsa-miR-511_st	0.062371316	0.035148563	-193.6293481
hsa-miR-512-3p_st	-4337.09573	0.115279602	0.008895696
hsa-miR-512-5p_st	-27.82693424	0.023284624	0.000826821
hsa-miR-513a-3p_st	-170.5064926	0.009319585	0.001229554
hsa-miR-513a-5p_st	-3.867094432	-3.597662051	-6.057740022
hsa-miR-513b_st	0.026895344	0.063845414	1.62611E-05
hsa-miR-513c_st	-9.871613058	0.005739275	-10.81775456
hsa-miR-514_st	0.002751107	0.001107592	-322.1482157

hsa-miR-514b-3p_st	0.001555238	0.003338644	0.000443481
hsa-miR-514b-5p_st	0.033471068	0.687054488	0.034608835
hsa-miR-515-3p_st	7.62312E-06	-2573.455052	-5.548205508
hsa-miR-515-5p_st	0.001589976	-2.875778416	-5.168871603
hsa-miR-516a-3p_st	-11.91726151	-79.74359379	-5.202562466
hsa-miR-516a-5p_st	-351.303366	-22.09217161	0.003718804
hsa-miR-516b_st	-42.00890204	-79.26861623	-7.399126872
hsa-miR-516b-star_st	-2092.760608	-147.7177035	-353.9786921
hsa-miR-517a_st	0.002678787	-1641.627036	-17.84284289
hsa-miR-517b_st	-119.9936719	-17.79065118	-4.73685843
hsa-miR-517c_st	0.175816264	0.197259007	0.046020761
hsa-miR-517-star_st	0.005366954	-457.2350867	-8.839416384
hsa-miR-518a-3p_st	0.01411641	0.080855776	0.036020813
hsa-miR-518a-5p_st	-36.2168306	-752.1545997	-157.9949493
hsa-miR-518b_st	-8.210442573	-162.8525333	-5.282624996
hsa-miR-518c_st	0.015376496	0.045314701	-7.061180635
hsa-miR-518c-star_st	0.106454397	0.044328355	0.023578677
hsa-miR-518d-3p_st	-7.870574191	0.016336419	-1892.99198
hsa-miR-518d-5p_st	-110.5785609	0.009542652	0.145714357
hsa-miR-518e_st	-3.48162051	-3.012591001	-28.78501324
hsa-miR-518e-star_st	-314.1031258	-386.0175926	-451.9431819
hsa-miR-518f_st	0.00092836	0.089723013	0.000136306
hsa-miR-518f-star_st	0.229235076	0.225826445	0.230014242
hsa-miR-519a_st	-227.3480951	-333.787259	-24.75691224
hsa-miR-519a-star_st	-1085.095575	0.075321705	-14.90182685
hsa-miR-519b-3p_st	-13.23772204	-34.83734195	6.03194E-06
hsa-miR-519b-5p_st	-17.13653414	-18.16946473	-3241.737687
hsa-miR-519c-3p_st	-10.26837037	-949.8117061	-12.02436623
hsa-miR-519c-5p_st	-55.43820631	0.000815759	0.000291726
hsa-miR-519d_st	0.021110346	-30.38125686	0.028011378
hsa-miR-519e_st	-75.99121125	0.007322995	-110.319711
hsa-miR-519e-star_st	-66.57747197	-14.98205203	-6.038819451
hsa-miR-520a-3p_st	0.012573698	0.0640629	-36.91865614
hsa-miR-520a-5p_st	0.233129637	0.002583435	-38.47378026
hsa-miR-520b_st	0.009557115	0.024048256	0.08842862
hsa-miR-520c-3p_st	0.032988549	0.153865162	0.043816537
hsa-miR-520c-5p_st	0.000155152	-35.20666947	0.046562519
hsa-miR-520d-3p_st	0.095043032	0.686983205	-17.11321905
hsa-miR-520d-5p_st	-24.8927215	-597.6796108	-35.09787672
hsa-miR-520e_st	0.004856069	0.006352568	-11.00984564
hsa-miR-520f_st	-154.6395107	-36.63075334	-7957.012384
hsa-miR-520g_st	0.009672722	-44.48002134	-11022.24427
hsa-miR-520h_st	-1251.30663	0.002690497	0.060792327
hsa-miR-521_st	-276.4676159	-30.87208656	0.020998328
hsa-miR-522_st	7.59861E-05	0.105743983	0.005425648
hsa-miR-522-star_st	0.010666965	-16.80771743	0.00379875
hsa-miR-523_st	-18.56546604	0.003874751	-18.09819012
hsa-miR-523-star_st	-196.4842314	5.73049E-05	-99.80428774
hsa-miR-524-3p_st	-571.4286077	-13.88326495	0.003372473
hsa-miR-524-5p_st	-31.82556043	-24.8584787	0.011060939
hsa-miR-525-3p_st	0.002226802	0.200067897	0.032327141

hsa-miR-525-5p_st	0.011805474	-11.10533559	0.004554158
hsa-miR-526a_st	-35.56471554	-24.90800468	0.011289806
hsa-miR-526b_st	-2.581146691	-7.95511103	-7.821914678
hsa-miR-526b-star_st	-2821.375058	0.049073104	0.00825763
hsa-miR-527_st	-7.727615009	-3.981464667	-11.49056136
hsa-miR-532-3p_st	-559.9393832	-6.285604528	0.143214185
hsa-miR-532-5p_st	0.365642135	-2.022923191	0.513350755
hsa-miR-539_st	0.001727899	-81.94586001	0.193991798
hsa-miR-541_st	-107.3520569	-5.450787315	-14.90234459
hsa-miR-541-star_st	-1849.845283	-23.25994464	-228.1144645
hsa-miR-542-3p_st	0.075695416	0.071037708	0.192919479
hsa-miR-542-5p_st	0.198804962	-18.43108963	0.250385148
hsa-miR-543_st	0.0606366	-1.360183761	0.043413265
hsa-miR-544_st	0.001360835	-267.4674878	-909.6529622
hsa-miR-544b_st	-964.4390116	-13625.18683	-59.85613302
hsa-miR-545_st	-1831.289107	0.020298983	0.098113659
hsa-miR-545-star_st	0.037872468	-24.32222528	0.004000246
hsa-miR-548a-3p_st	0.011501383	-2.525885147	-3.856439622
hsa-miR-548a-5p_st	0.004915693	-5.997833978	0.054909143
hsa-miR-548b-3p_st	0.002993129	-99.97400507	-7.165043218
hsa-miR-548b-5p_st	0.000241834	0.015538246	-37.86492901
hsa-miR-548c-3p_st	-132.9846537	-17.74197586	-37.71323738
hsa-miR-548c-5p_st	0.023779182	0.069098797	0.000849577
hsa-miR-548d-3p_st	-231.6697131	-56.12729487	-1055.716796
hsa-miR-548d-5p_st	-11.8613032	0.060254784	0.000156938
hsa-miR-548e_st	0.006686987	0.006442631	0.000389786
hsa-miR-548f_st	-2.631962347	-2.197765564	-4.00321794
hsa-miR-548g_st	-10.40824452	-3.917436526	0.00095651
hsa-miR-548h_st	-19.93892524	-45.87232429	0.01524941
hsa-miR-548i_st	-97.12357933	-8.367267494	-11.16292123
hsa-miR-548j_st	0.088522018	0.246385659	0.397312757
hsa-miR-548k_st	-431.3456836	-4.25086315	0.013367869
hsa-miR-548l_st	0.053384103	0.466583942	0.003368874
hsa-miR-548m_st	0.023789515	0.371445148	0.341885784
hsa-miR-548n_st	0.103360643	-106.8046909	-784.0125442
hsa-miR-548o_st	-8829.023694	0.030342679	-70.5269613
hsa-miR-548p_st	0.050697926	0.010370164	0.08192332
hsa-miR-548q_st	0.016931995	0.009792686	-669.8741271
hsa-miR-548s_st	0.08373818	-2723.301118	-47.81832266
hsa-miR-548t_st	0.039579511	0.024031045	0.02713449
hsa-miR-548u_st	0.00224297	0.000212883	-179.6532279
hsa-miR-548v_st	0.067162351	-17.98912415	-184.3573235
hsa-miR-548w_st	0.113131995	-15.0985926	-942.5935882
hsa-miR-548x_st	-1154.960302	-2.244355268	-33.85675502
hsa-miR-549_st	-142.3649686	-349.3232541	0.009204675
hsa-miR-550_st	-1963.857982	0.161544509	0.216784757
hsa-miR-550-star_st	0.175431134	-134.0895238	0.034295706
hsa-miR-551a_st	-7.324780621	-27.30891741	-17.29532643
hsa-miR-551b_st	0.002562182	-6.204360858	0.142288139
hsa-miR-551b-star_st	0.010219289	-13.60432155	0.042301794
hsa-miR-552_st	-112.7088792	-4.466010152	-10.55895189

hsa-miR-553_st	0.005139098	1.114677743	0.32165572
hsa-miR-554_st	0.011585186	-50.96651504	0.061327551
hsa-miR-555_st	0.01470896	0.000891381	-34.45942893
hsa-miR-556-3p_st	0.012075482	-5740.949835	-37.18591822
hsa-miR-556-5p_st	0.004003536	-64.3433917	-69.06120008
hsa-miR-557_st	0.070597553	0.716322711	0.290270419
hsa-miR-558_st	0.222578256	-195.5402105	0.099741325
hsa-miR-559_st	-4565.376187	0.02097906	0.042624286
hsa-miR-561_st	-17.7669625	-6.628235803	-24.33626566
hsa-miR-562_st	-11.00305386	-2.479462182	-8.628933215
hsa-miR-563_st	-5.346838487	-5.553515164	-23.00649036
hsa-miR-564_st	0.213779081	2.91556E-06	0.135002233
hsa-miR-566_st	0.001203327	-11.76862871	0.030677523
hsa-miR-567_st	0.068660245	-48.27759521	-73.50424564
hsa-miR-568_st	-604.785217	-18.29464511	0.002338545
hsa-miR-569_st	-79.33994525	0.006694021	-22759.85436
hsa-miR-570_st	-345.2396619	0.012132251	-29.49853353
hsa-miR-571_st	-152.4496661	-10.02136475	0.004481968
hsa-miR-572_st	0.36873556	-4.517030816	0.228511725
hsa-miR-573_st	-668.6794309	-17.20519583	-366.9940253
hsa-miR-574-3p_st	-0.329322405	-11.0897346	-0.391862307
hsa-miR-574-5p_st	-0.219048118	-14.96299136	-0.184363606
hsa-miR-575_st	-6.511292484	-7.301286344	-115.5445032
hsa-miR-576-3p_st	-44.56409289	-7.858510024	-14.48365555
hsa-miR-576-5p_st	0.045595701	0.000916485	-69.73528538
hsa-miR-577_st	0.018634434	-11.54811163	0.002182899
hsa-miR-578_st	-5.338626153	-3.908635549	-4.849842121
hsa-miR-579_st	-13.47978772	-14.14035893	-105.2662757
hsa-miR-580_st	-3740.342422	-3.56613974	-14.44174451
hsa-miR-581_st	0.331679815	0.398151535	0.672334402
hsa-miR-582-3p_st	-46.20846197	-4.095268686	-61.03944801
hsa-miR-582-5p_st	0.052809189	-38.32340064	0.102481296
hsa-miR-583_st	-190.9100745	-5.223092898	-21559.66486
hsa-miR-584_st	0.824160756	0.044628886	1.055041231
hsa-miR-585_st	-23.04516183	-5.108820935	-8.748431808
hsa-miR-586_st	-62.81355496	-55.9398045	-61.42130971
hsa-miR-587_st	0.043739749	0.057030933	0.066496679
hsa-miR-588_st	-72.10351948	-33.66647011	0.012901552
hsa-miR-589_st	0.01833817	-686.2040628	-102.8985266
hsa-miR-589-star_st	-102.7358881	-5.495692649	0.038669453
hsa-miR-590-3p_st	0.272240281	-3.323066712	0.025742438
hsa-miR-590-5p_st	-143.2969846	-588.936599	0.011201259
hsa-miR-591_st	-29.9765775	-51.22253739	-47.80972647
hsa-miR-592_st	-131847.5694	0.00722857	0.090259086
hsa-miR-593_st	0.080206771	0.442076053	0.238562888
hsa-miR-593-star_st	-281.0942191	-4.528727307	-7.209224171
hsa-miR-595_st	-39.90347664	1.763916016	-200.9177359
hsa-miR-596_st	0.073919822	0.011600906	-141.0860711
hsa-miR-597_st	-105.4046553	-5.953660013	-1.770647888
hsa-miR-598_st	0.002714983	-21.28755924	-28.51983903
hsa-miR-599_st	-236.0178058	0.012388914	0.028331959

hsa-miR-600_st	0.001172891	0.057214248	0.009314277
hsa-miR-601_st	-14.09040278	1.563737745	-18.57066675
hsa-miR-602_st	0.991038169	-2.010898607	1.116377145
hsa-miR-603_st	0.064719614	-13.80199452	-115.4538901
hsa-miR-604_st	-56.91680797	-7.416809544	-52.09390813
hsa-miR-605_st	-36.08511062	-187652467.6	-153.6234292
hsa-miR-606_st	0.113632365	-23.92929274	0.073148612
hsa-miR-607_st	0.144098058	-5.244645796	0.250392154
hsa-miR-608_st	-0.773031562	0.867866449	-0.157252731
hsa-miR-609_st	0.049466653	0.557865585	0.030459848
hsa-miR-610_st	-4.979015838	0.508387538	-6.279856546
hsa-miR-611_st	-0.52322472	-13.42324972	-1.259710975
hsa-miR-612_st	-1615.27653	0.955610845	0.006717442
hsa-miR-613_st	-23394.30607	3.58981E-05	0.055522911
hsa-miR-614_st	-42.73313495	-81.06508219	0.000407818
hsa-miR-615-3p_st	-57.59088134	-24.89098284	0.052119977
hsa-miR-615-5p_st	-13.20287232	0.165546452	-2.629248779
hsa-miR-616_st	-7.999746153	0.253133256	0.000433993
hsa-miR-616-star_st	0.008747861	0.053395425	-13.78810925
hsa-miR-617_st	-24.26319695	0.097216434	-30.91329581
hsa-miR-618_st	-6.629942596	-4.639827003	-8.887335923
hsa-miR-619_st	-12.18056347	-15.19088841	-2.445768471
hsa-miR-620_st	-178.3861496	-5.315241809	0.001384361
hsa-miR-621_st	-35.97438168	-22.79977228	-30.84071985
hsa-miR-622_st	0.003907563	0.287262233	-437.1459695
hsa-miR-623_st	-23.75200721	-30.90865565	-18.91345829
hsa-miR-624_st	-7.657040727	-25.40869361	-193.9395726
hsa-miR-624-star_st	-38757.70659	-769.6795368	-160.2652179
hsa-miR-625_st	0.634515799	-11.91117512	1.109420877
hsa-miR-625-star_st	0.077409429	0.092000596	0.03139984
hsa-miR-626_st	-398304.775	-10.77939493	-481.614026
hsa-miR-627_st	0.016619722	-9.285265057	-57.05618865
hsa-miR-628-3p_st	0.011375609	0.023026849	0.287685804
hsa-miR-628-5p_st	0.015053204	-35.51132796	-10.10374934
hsa-miR-629_st	0.969489345	-19.87391226	1.205239492
hsa-miR-629-star_st	-2.347405288	-2.609616001	-5.775102402
hsa-miR-630_st	-28849.4913	-18.37818847	-52.5979845
hsa-miR-631_st	-7.044379734	-29.91103542	-63.3839396
hsa-miR-632_st	0.184210923	0.486276102	0.031066178
hsa-miR-633_st	0.006518867	-91.35605977	-27.41167281
hsa-miR-634_st	0.019160096	-97.59140221	0.067528259
hsa-miR-635_st	-1.50965107	-80.57609712	-28.05511946
hsa-miR-636_st	0.323709792	0.041587649	0.267872494
hsa-miR-637_st	0.251755571	1.790134286	0.560775574
hsa-miR-638_st	0.289411721	0.001235171	0.178442881
hsa-miR-639_st	0.058632506	0.015136258	0.448275108
hsa-miR-640_st	0.020599856	-2.4275495	-14.95483364
hsa-miR-641_st	0.085084806	-257.2703726	0.055621685
hsa-miR-642_st	-25.38002849	-362.2802522	-91.30630858
hsa-miR-643_st	-554.1546526	3.93004E-05	-10.99419019
hsa-miR-644_st	-90.65113979	-461.0622313	-1678.005104

hsa-miR-645_st	-33.71340128	0.003199543	-207.9971474
hsa-miR-646_st	0.22784202	0.006485081	0.275374107
hsa-miR-647_st	-4.775662892	0.035563171	-83.84231394
hsa-miR-648_st	0.25509722	0.011582603	0.062568769
hsa-miR-649_st	0.086921781	0.205711684	0.006769916
hsa-miR-650_st	-822.4246246	-2.258248611	0.027418227
hsa-miR-651_st	-120.7411132	-17.55212615	0.061432349
hsa-miR-652_st	0.114499333	-4.165364805	0.117200441
hsa-miR-653_st	-26.30024116	-2.185032889	-45.6886391
hsa-miR-654-3p_st	0.061427888	-1.990427921	0.256524016
hsa-miR-654-5p_st	0.028490739	-2.826763516	0.207191563
hsa-miR-655_st	0.018671309	0.001070664	0.28437156
hsa-miR-656_st	0.002572721	-3.625479466	-316.5444052
hsa-miR-657_st	-6.86705853	-9.779647652	0.002507506
hsa-miR-658_st	-745.5560652	1.92936267	-1.367011587
hsa-miR-659_st	-8.295867972	0.11653416	-16.50676902
hsa-miR-660_st	0.024653239	-2.173070294	0.166570097
hsa-miR-661_st	0.007910413	0.058013058	-0.161430475
hsa-miR-662_st	0.106381324	0.039364941	0.005281365
hsa-miR-663_st	-128467.0033	0.028762464	-51.92886417
hsa-miR-663b_st	0.074711749	-29.36123062	-0.405524058
hsa-miR-664_st	0.094681136	4.50241E-05	0.003173013
hsa-miR-664-star_st	0.025702983	0.08298807	0.120948494
hsa-miR-665_st	-81.56810804	-1.707688472	-34.22977686
hsa-miR-668_st	-4.239605945	-4.396232986	-4.57089443
hsa-miR-670_st	-42.66198991	0.215085396	-279.7558728
hsa-miR-671-3p_st	0.316282512	-8.837288039	0.260920726
hsa-miR-671-5p_st	-13.61210251	-2.078145824	0.047467119
hsa-miR-675_st	0.041583366	0.447073432	0.59074596
hsa-miR-675-star_st	0.465679314	-858.7764639	0.475136558
hsa-miR-7_st	0.059276424	0.033584411	0.029008221
hsa-miR-708_st	0.012823071	0.037385576	0.161110721
hsa-miR-708-star_st	0.004049222	-4.516032563	-11.9018079
hsa-miR-711_st	-31.60888962	0.197529136	0.049522494
hsa-miR-718_st	-0.417583459	0.06031322	-10.51513656
hsa-miR-7-1-star_st	0.085620905	-35.28283224	0.040110476
hsa-miR-720_st	0.008439362	-8.13869119	0.989067047
hsa-miR-7-2-star_st	0.004759206	0.461574848	-50.06359856
hsa-miR-744_st	-3.704997625	-45.68616861	0.00063363
hsa-miR-744-star_st	-296.6102113	-98.94147833	-111.0182448
hsa-miR-758_st	0.354835854	-19.62579277	0.699181941
hsa-miR-759_st	-12973.59791	-228.1937274	-28.31774161
hsa-miR-760_st	0.137016575	4.759225849	1.14674114
hsa-miR-761_st	0.019720866	-9.233983718	0.039675063
hsa-miR-762_st	0.14063625	0.1935912	0.183252486
hsa-miR-764_st	-63.90896124	0.212323738	-12.43714752
hsa-miR-765_st	-58.94693219	1.59608607	0.000217091
hsa-miR-766_st	0.05395005	0.047582006	-7395.245666
hsa-miR-767-3p_st	-17.05806249	-25.19159426	-10.16391337
hsa-miR-767-5p_st	-7.270469326	-3.238706332	-31.40726899
hsa-miR-769-3p_st	0.28523664	0.375364529	0.477362846

hsa-miR-769-5p_st	2.423258849	0.177819083	3.009853721
hsa-miR-770-5p_st	0.04782794	1.15634E-05	0.001241822
hsa-miR-802_st	0.00405215	0.047084877	0.003108174
hsa-miR-873_st	0.004965246	-37.65771449	3.07476E-06
hsa-miR-874_st	0.024479575	-11.71206925	0.00563558
hsa-miR-875-3p_st	0.022867488	-16.68205116	-139.9616614
hsa-miR-875-5p_st	0.001908204	-10.01077727	0.002015112
hsa-miR-876-3p_st	0.014445756	0.041582551	0.104587237
hsa-miR-876-5p_st	-132.7870442	-194.5812609	-16.63120226
hsa-miR-877_st	-2.527407291	-12.32799876	-31.91209581
hsa-miR-877-star_st	-5.404117822	0.000414835	0.001478749
hsa-miR-885-3p_st	0.416177879	0.194275548	0.342193411
hsa-miR-885-5p_st	0.289421404	0.007320171	0.184927951
hsa-miR-886-3p_st	0.113238644	0.018708495	0.218974395
hsa-miR-886-5p_st	-3.426556356	-4.346559907	-10.52451964
hsa-miR-887_st	-1.771274784	-1.103488007	0.001384621
hsa-miR-888_st	-365.8227368	-5.583620145	-12.91748721
hsa-miR-888-star_st	0.001595883	-15.04878929	-8.814136233
hsa-miR-889_st	0.02530072	-47.65835143	0.1213007
hsa-miR-890_st	-16.31583217	-13.34022794	-9.85352476
hsa-miR-891a_st	-72.82834236	0.015684557	-411.4980942
hsa-miR-891b_st	-11.22490368	-17.19520898	-11.20169832
hsa-miR-892a_st	-13.42147943	-235.5182245	-6.917050514
hsa-miR-892b_st	-4.315374607	-3.103204585	-22.08199897
hsa-miR-9_st	0.041833339	-13.23565124	-37.99317079
hsa-miR-920_st	0.172814388	0.047232981	0.29456128
hsa-miR-921_st	-3.708373137	0.072974539	-14.60099989
hsa-miR-922_st	0.023677977	-27.22434416	0.284397691
hsa-miR-924_st	0.063616946	0.556210182	0.086485105
hsa-miR-92a_st	-145.1239773	-2.490477465	0.091028924
hsa-miR-92a-1-star_st	-130.2443243	-3.252792992	-48.06129329
hsa-miR-92a-2-star_st	0.052948541	0.591294099	0.061452923
hsa-miR-92b_st	-42.02524347	-2015.50589	0.511816476
hsa-miR-92b-star_st	0.069172945	-10.50631087	0.013737246
hsa-miR-93_st	-8.924811414	6.55857E-05	-173.9577957
hsa-miR-933_st	0.00603426	-0.748876347	0.042430232
hsa-miR-934_st	-162.3383552	-2.083380382	-42.13005474
hsa-miR-935_st	0.27730756	-30.33976956	0.336060206
hsa-miR-936_st	0.075018197	1.468567092	0.042239087
hsa-miR-937_st	0.036228745	0.227393552	0.072521413
hsa-miR-938_st	0.014435061	0.058371043	0.057899188
hsa-miR-939_st	0.554504367	1.983932816	0.940388879
hsa-miR-93-star_st	-50.06678677	-0.915586256	0.02714767
hsa-miR-940_st	-3.412676036	-1.715815781	-3.607858058
hsa-miR-941_st	-861.9056166	-174.8421966	-109.20428
hsa-miR-942_st	0.0516737	0.010530454	0.064482476
hsa-miR-943_st	0.098489897	-1.105510076	0.102960445
hsa-miR-944_st	0.034606975	-95.90144912	0.000437374
hsa-miR-95_st	-2477.527646	-3.059929884	-10.01689314
hsa-miR-96_st	-5.045104646	0.043156739	-13.86269953
hsa-miR-96-star_st	-10.83356939	0.010781811	-41.6388886

hsa-miR-98_st	0.119647502	0.025745165	0.138904172
hsa-miR-99a_st	0.247775968	-1.028770278	0.100030824
hsa-miR-99a-star_st	0.08732025	-70.30478741	-230.6610068
hsa-miR-99b_st	-1.86842538	-73.62286395	-7.76969686
hsa-miR-99b-star_st	-6.429657722	-19.02773063	-311.9212262
hsa-miR-9-star_st	-17.67847569	-4.26409294	-21626.29758
hsv1-miR-H1_st	-8.523002734	0.656523698	-6.001890192
hsv1-miR-H11_st	-15.87055082	-10.33546011	-18.05845017
hsv1-miR-H12_st	0.003109512	-20.4796977	0.010684943
hsv1-miR-H13_st	0.052739811	-14.07993612	0.033006532
hsv1-miR-H14-3p_st	0.000883813	-0.673511495	-8.388194157
hsv1-miR-H14-5p_st	-2.760704113	-2.013418917	-1.572246534
hsv1-miR-H15_st	-12.25830359	-1.696987265	-42.51579593
hsv1-miR-H16_st	0.11886256	0.656628226	0.201817476
hsv1-miR-H17_st	0.232700112	0.04850786	0.092546766
hsv1-miR-H18_st	-44.1408238	-0.787767499	-7.240158698
hsv1-miR-H1-star_st	-8.10173776	-14.35639906	-12.69468523
hsv1-miR-H2_st	-22.98245273	0.098170989	-125.8066854
hsv1-miR-H2-star_st	0.045229805	0.170481868	-2.020095002
hsv1-miR-H3_st	0.171130315	0.904638766	-34.16658061
hsv1-miR-H3-star_st	-56.12393106	-20.07081105	-2785.602155
hsv1-miR-H4_st	0.034698189	0.088898491	0.227782357
hsv1-miR-H4-star_st	0.076275669	-663.4537141	0.044284994
hsv1-miR-H5-3p_st	-5.174258	-8.955632715	-4.469551503
hsv1-miR-H5-5p_st	0.937814054	0.054805385	0.464606506
hsv1-miR-H6-3p_st	0.539575455	-32.87718215	-27.53710494
hsv1-miR-H6-5p_st	0.035398353	1.303647867	-33.265725
hsv1-miR-H7_st	-53.32292716	-45.95227577	-1146.062817
hsv1-miR-H7-star_st	-11.45329881	-8.18151158	-13.48691722
hsv1-miR-H8_st	-2025.405061	2.09673296	0.00099875
hsv1-miR-H8-star_st	0.00113165	-18.60905953	0.001517843
hsv2-miR-H10_st	0.340658996	0.035654881	0.217930249
hsv2-miR-H11_st	0.260844622	0.099688906	0.007838535
hsv2-miR-H11-star_st	-12.24698093	-3.293982114	-14.73198215
hsv2-miR-H12_st	-15.07491842	-147.0271296	-21.5558453
hsv2-miR-H13_st	0.011748609	-2.397386288	0.000307652
hsv2-miR-H19_st	0.048988961	0.000987939	-998.3435474
hsv2-miR-H2_st	0.024072453	0.031232079	-246.1055316
hsv2-miR-H20_st	0.16208515	0.001780671	0.017136381
hsv2-miR-H21_st	-25.88073295	-2.545545278	-19.37060589
hsv2-miR-H22_st	0.074037594	2.333929926	0.000136913
hsv2-miR-H23_st	0.348540279	0.000999382	0.238006404
hsv2-miR-H23-star_st	-145.4777736	-22.64380873	-75.83511684
hsv2-miR-H24_st	-0.152858515	-0.406950588	-0.000524878
hsv2-miR-H25_st	0.025901684	0.045290224	-34.89498727
hsv2-miR-H3_st	0.063988256	6.844745749	0.137904907
hsv2-miR-H4-3p_st	-57.87998084	-759.1129469	0.0058822
hsv2-miR-H4-5p_st	0.062044832	0.071954307	0.05089739
hsv2-miR-H5_st	0.000228055	-26.57674333	-44.23599537
hsv2-miR-H6_st	-0.431864904	-1.029624363	-0.347379523
hsv2-miR-H6-star_st	0.000800154	0.576883542	0.115254139

hsv2-miR-H7-3p_st	-17.35785636	-104.3595544	-175.1894984
hsv2-miR-H7-5p_st	-21.92675737	-59.83761382	-95.10441265
hsv2-miR-H9-3p_st	-25.12874513	-946.8620954	-50.54920753
hsv2-miR-H9-5p_st	0.09501498	1.032548629	0.090840453
