# Supplementary material for: Human Fibroblast Reprogramming to Pluripotent Stem Cells Regulated by the miR19a/b-PTEN Axis
Source: PLoS One. 2014 Apr 16;9(4):e95213. doi: 10.1371/journal.pone.0095213 (PMC3989277; doi:10.1371/journal.pone.0095213)
Supplement: Table S2 — All the primers we used. (PDF) [file pone.0095213.s006.pdf]

Table S2.

primers for real-time PCR

PTEN_fwd	CACAATTCCCAGTCAGAGGCGC
PTEN_rev	GCTGGCAGACCACAAACTGAGGA

reverse-transcript PCR primers for endogenous

endoOct4_fwd	CCTCACTTCACTGCACTGTA
endoOct4_rev	CAGGTTTTCTTTCCCTAGCT
endoNanog_fwd	TGAACCTCAGCTACAAACAG
endoNanog_rev	TGGTGGTAGGAAGAGTAAAG
endoSox2_fwd	CCCAGCAGACTTCACATGT
endoSox2_rev	CCTCCCATTTCCCTCGTTTT
endoRex1_fwd	TCGCTGAGCTGAAACAAATG
endoRex1_rev	CCCTTCTTGAAGGTTTACAC

reverse-transcript PCR primers for retroviral silencing

Tg-pMX_fwd	GGGTGGACCATCCTCTAGAC
Tg-hOct4	CCAGGTCCGAGGATCAAC
Tg-hSox2	GGGCTGTTTTTCTGGTTG
Tg-hKlf4	GGAAGTCGCTTCATGTGG
Tg-hc-Myc	CCTCGTCGCAGTAGAAATAC

primers for CDS amplifying

PTEN_S	GCGGATCCATGACAGCCATCATCAAAG
PTEN_AS	GCGAATTCTCAGACTTTTGTAATTTGTG

miRNA specific primers for real-time PCR

RT-U6	ATGGAACGCTTCACGAAT
U6-fwd	GCTTCGGCAGCACATATACTAAAAT
U6-rev	CGCTTCACGAATTTGCGTGTCAT
RT-7a	GTCGTATCCAGTGCGTGTCGTGGAGTCGGCAATTGCACTGGATACGACAACTAT
L7aF	GGGCCCGTGAGGTAGTAGGTT
RT-7e	GTCGTATCCAGTGCGTGTCGTGGAGTCGGCAATTGCACTGGATACGACAACTAT
L7eF	GGCGCCTGAGGTAGGAGGTT
RT-302a	GTCGTATCCAGTGCGTGTCGTGGAGTCGGCAATTGCACTGGATACGACTCACCTT
F302a	GGGCCCTAAGTGCTTCCATGT
RT-367	GTCGTATCCAGTGCGTGTCGTGGAGTCGGCAATTGCACTGGATACGACACTCCAT
367F	GGGCCCAATTGCACTTTAGCA
RT-17	GTCGTATCCATGGCGTGTCGTGGAGTCGGCAATTGCCATGGATACGACCTACCTG
17F	GGCCCAAAGTGCTTACAGTGC
RT-20b/a	GTCGTATCCATGGCGTGTCGTGGAGTCGGCAATTGCCATGGATACGACCTACCTG
F20a	CCGGTAAAGTGCTTATAGTGC
RT-18a-5p	GTCGTATCCATGGCGTGTCGTGGAGTCGGCAATTGCCATGGATACGACCTATCT
F18a	CCGGTAAGGTGCATCTAGTGC
RT-19a=RT-19b	GTCGTATCCAGTGCGTGTCGTGGAGTCGGCAATTGCACTGGATACGAGTCAGTT
F19a	GGCCTGTGCAAATCTATGC

RT19b	GTCGTATCCAGTGCGTGTCGTGGAGTCGGCAATTGCACTGGATACGAGTCAGTT
F19b	GGGTGTGCAAATCCATGCA
RT-92a	GTCGTATCCAGTGCGTGTCGTGGAGTCGGCAATTGCACTGGATACGACACAGGC
F92a	CCGGTATTGCACTTGTCCCG
UP	TCCATGGCGTGTCGTGGAGT
RT-20b	GTCGTATCCAGTGCGTGTCGTGGAGTCGGCAATTGCACTGGATACGACCTACCT
F20b	CCGGCAAAGTGCTCATAGTGC
RT-146a	GTCGTATCGAGTGCAGGGTCCGAGGTATTCGCACTCGATACGACAACCCA
F-146a	GGCCTGAGAACTGAATTCCA
universal primer	TGCGTGTCGTGGAGTC
universal primer 17	TCCATGGCGTGTCGTGGAGTC
